# Spatiotemporal analysis of urban expansion and environmental impacts in five Saudi Arabian cities by utilizing geospatial and remote sensing approach

**DOI:** 10.1371/journal.pone.0354154

**Published:** 2026-09-01

**Authors:** Ahmed Ali A. Shohan, Abdulla Al Kafy, Saleh Alsulamy, Md Tanvir Miah, Jannatun Nahar Fariha, Md Hojaifa Al Hossain Khan, Abdullah Al Baky

**Affiliations:** 1 Architecture Department, College of Architecture and Planning, King Khalid University, KSA, Asser, Kingdom of Saudi Arabia; 2 Department of Geography and the Environment, The University of Texas at Austin, Austin, Texas, United of States of America; 3 Urban and Rural Planning Discipline, Khulna University, Khulna, Bangladesh; 4 Department of Geoscience, Texas Tech University, Texas, United of States of America; 5 Department of English, Bangladesh Army International University of Science and Technology, Cumilla, Bangladesh; Central University of Rajasthan, INDIA

## Abstract

Rapid urbanization in Saudi Arabia has fundamentally reshaped metropolitan landscapes, creating unprecedented challenges for environmental sustainability in arid regions. This study presents a spatiotemporal analysis of urban expansion patterns and associated environmental impacts across five major Saudi cities (Riyadh, Makkah, Jeddah, Madinah, and Dammam) over a 30-year period (1993–2023) using remote sensing and geographic information systems (GIS). We employed supervised classification of multi-temporal Landsat imagery, integrating spectral indices such as NDVI, SAVI, and NDWI to enhance land-cover discrimination. Classification accuracies ranged from 92% to 95.2% and were validated through stratified sampling and error matrix analysis. Post-classification change detection algorithms quantified urban growth dynamics, while binary logistic regression models assessed topographic influences on spatial development patterns. Results reveal dramatic urban transformation across all study cities. Madinah experienced the largest absolute expansion (759.2 km²), followed by Riyadh (648.08 km²) and Jeddah (640.12 km²). Growth rates varied significantly: Riyadh exhibited the highest proportional increase (236.8%), while Makkah showed more constrained expansion (48.92 km²) due to topographic limitations. Spatial analysis identified distinct urbanization patterns, with centralized growth in Makkah and Madinah versus dispersed, sprawling development in Riyadh, Jeddah, and Dammam. Topographic analysis showed that slope significantly influenced urban development in Jeddah (p = 0.003) and Makkah (p = 0.030), while elevation significantly shaped expansion patterns in Riyadh, Jeddah, Makkah, and Madinah (p < 0.05). Model fit statistics (Nagelkerke R² = 0.06–0.24; ROC AUC = 0.56–0.72) indicate that topographic variables provide modest but meaningful explanatory power, with additional socioeconomic variables needed for comprehensive modeling. These findings reveal complex relationships among urban growth, topographic constraints, and environmental pressures in arid metropolitan areas. The observed urban expansion patterns are consistent with documented effects of government policies, oil-driven economic growth, and population migration on Saudi urban landscapes, with potential implications for habitat continuity, local environmental quality, and resource pressures. This research provides baseline data for evidence-based urban planning and offers a replicable methodological approach for monitoring rapid urbanization in similar arid environments.

## 1. Introduction

The unprecedented pace of global urbanization in the 21st century has fundamentally transformed landscapes worldwide, with urban populations expected to reach 68% by 2050 [1]. This transformation is particularly pronounced in arid and semi-arid regions, where rapid economic development and population growth have created unique challenges for sustainable environmental management. The Arabian Peninsula exemplifies this phenomenon, where oil-driven economic prosperity has catalyzed dramatic urban expansion across desert landscapes, fundamentally altering ecological systems and environmental dynamics [2].

Urban expansion in arid environments poses distinct challenges compared with temperate regions, given inherent constraints such as water scarcity, extreme temperatures, and fragile desert ecosystems [3]. The Kingdom of Saudi Arabia, the largest economy in the Gulf Cooperation Council, has experienced one of the world’s most rapid urbanization rates over the past three decades, with the urban population rising from 77% in 1990 to over 84% by 2020. This transformation has been driven primarily by government-led development initiatives, substantial oil revenues, and strategic economic diversification policies [4]. The spatial and temporal dynamics of urban growth in arid regions require sophisticated analytical approaches that capture both the magnitude and environmental implications of landscape transformation. Remote sensing (RS) and geographic information systems (GIS) have become indispensable tools for monitoring urban expansion, providing consistent, multi-temporal data essential for understanding spatiotemporal patterns of land use change [5, 6]. Satellite-based change detection techniques offer particular advantages in arid regions, where cloud-free conditions and distinct spectral signatures between urban and natural surfaces facilitate accurate classification and monitoring [7, 8].

Landsat satellite imagery, with its 30-meter spatial resolution and multi-decadal temporal coverage, provides an optimal platform for analyzing urban expansion dynamics at metropolitan scales. The transition from Landsat Thematic Mapper (TM) to Operational Land Imager (OLI) sensors has enhanced spectral capabilities while maintaining the temporal continuity essential for long-term change detection studies [7]. However, differences in radiometric resolution and spectral characteristics between TM (7-bit) and OLI (12-bit) sensors require careful consideration in multi-temporal analysis to ensure classification consistency and accuracy [8]. Methodological advances in urban change detection have increasingly emphasized integrating spectral indices with traditional band combinations to improve classification accuracy in arid environments. The Normalized Difference Vegetation Index (NDVI), Soil Adjusted Vegetation Index (SAVI), and Normalized Difference Water Index (NDWI) have proven particularly effective at discriminating against urban surfaces from natural desert features [9–11]. These indices enhance the spectral separability of land cover classes, addressing challenges posed by the spectral similarity between built surfaces and natural desert materials.

The environmental implications of rapid urbanization in arid regions extend beyond simple land-cover conversion, encompassing complex interactions among urban development patterns, topographic constraints, and ecosystem dynamics. Topographic factors, particularly elevation and slope, play crucial roles in shaping urban expansion patterns, influencing both development feasibility and environmental impacts [12]. Understanding these relationships requires quantitative modeling approaches that can identify significant drivers of urban growth while accounting for spatial heterogeneity and environmental constraints. Binary logistic regression modeling has emerged as a powerful tool for analyzing the probabilistic relationships between topographic variables and urban development patterns. This approach enables researchers to quantify the relative influence of elevation, slope, and other environmental factors on urbanization processes, providing insights essential for evidence-based planning and environmental management [13]. Such analyses are particularly relevant in mountainous arid regions, where topographic constraints significantly influence development feasibility and environmental sustainability.

Saudi Arabia’s five largest metropolitan areas, Riyadh, Makkah, Jeddah, Madinah, and Dammam, represent diverse geographic settings and urbanization drivers, providing an ideal natural laboratory for studying arid urban expansion. Previous research has documented significant urban expansion across these cities, with studies indicating substantial land cover changes over the past three decades [2,14]. However, a comprehensive comparative analysis that incorporates topographic influences, environmental impacts, and spatiotemporal patterns across all five cities remains limited. Most existing studies have focused on individual cities or shorter temporal periods, limiting understanding of regional urbanization dynamics and comparative development patterns. The environmental consequences of rapid urban expansion in Saudi Arabia have become increasingly apparent, with studies documenting habitat fragmentation, biodiversity loss, and ecosystem degradation across metropolitan regions [3]. Air quality deterioration, particularly in industrial areas around Dammam and transportation corridors in Riyadh, has raised concerns about public health and environmental sustainability [15]. Urban heat island effects, exacerbated by extensive concrete surfaces and reduced vegetation cover, compound existing thermal stress in these already extreme climate environments. Water resource constraints represent perhaps the most critical environmental challenge facing Saudi urban areas. Rapid population growth and urban expansion have dramatically increased water demand, straining groundwater resources and necessitating costly desalination and water-transfer projects. The spatial patterns of urban development directly influence water infrastructure requirements and environmental impacts, highlighting the importance of understanding urbanization dynamics for sustainable resource management. Contemporary approaches to urban sustainability assessment increasingly emphasize integrating RS data with environmental indicators to support evidence-based planning and policy formulation [16]. This integration enables researchers to quantify relationships between urban expansion patterns and environmental outcomes, providing crucial information for developing mitigation strategies and sustainable development policies. Such approaches are particularly relevant in arid regions, where environmental constraints and resource limitations demand careful consideration of development impacts.

The present study addresses critical knowledge gaps in understanding arid urban expansion by providing a spatiotemporal analysis of urbanization patterns and environmental impacts across Saudi Arabia’s five largest metropolitan areas. By integrating multi-temporal Landsat imagery, change detection techniques, and quantitative modeling, this research aims to: (1) quantify urban expansion patterns and rates across five major Saudi cities from 1993 to 2023; (2) analyze topographic influences on urban development using binary logistic regression; (3) assess the environmental implications of observed urbanization patterns; and (4) provide evidence-based recommendations for sustainable urban planning in arid environments. This research contributes to the growing body of literature on arid urban geography while advancing methods for monitoring rapid urbanization in resource-constrained environments. The findings have immediate relevance for urban planning and environmental management in Saudi Arabia and broader applicability to similar arid regions experiencing rapid development pressures worldwide.

## 2. Materials and methods

### 2.1. Study area

**Fig 1:**
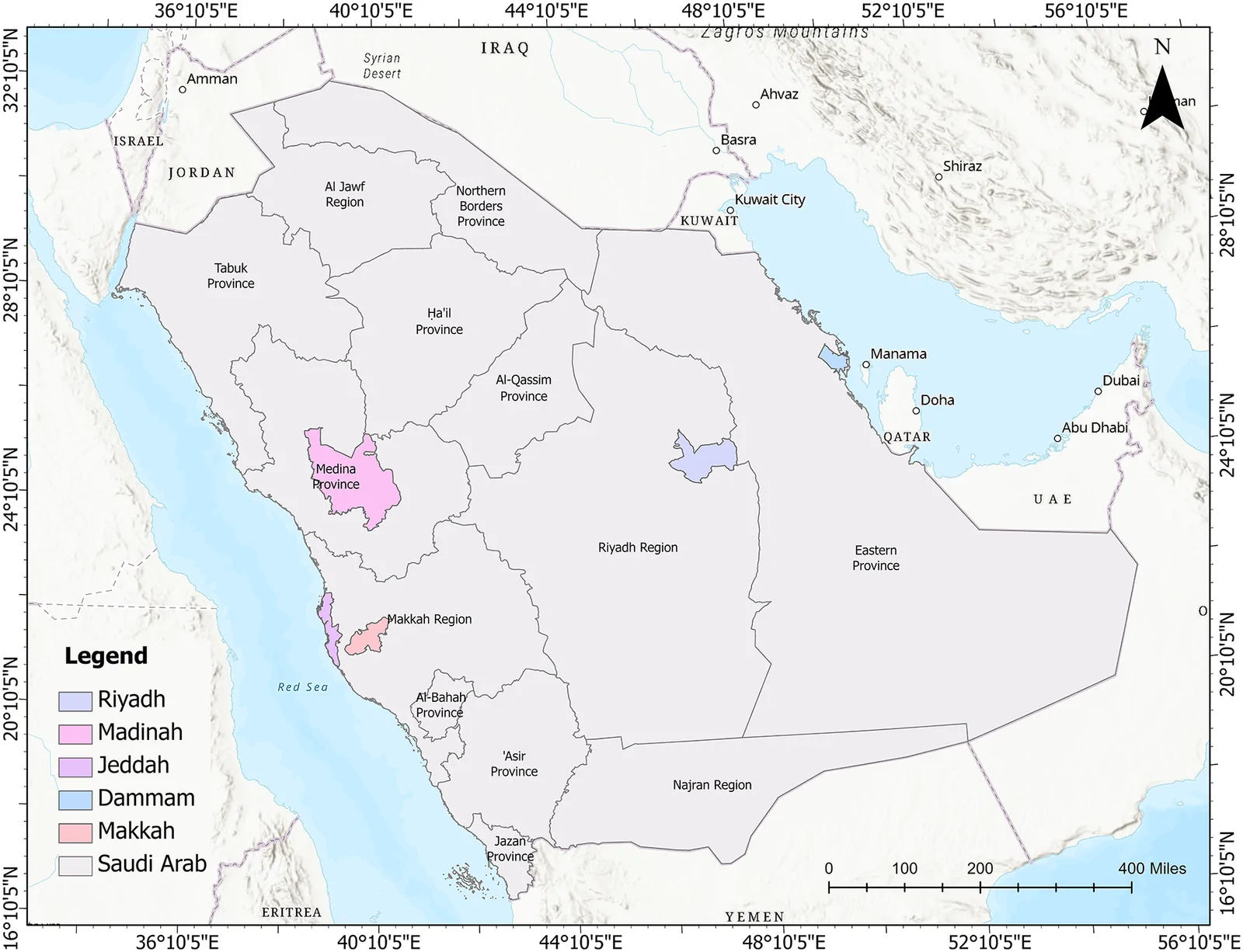
Geographic location of the five study cities within Saudi Arabia. Administrative boundaries © NextGIS. No copyrighted basemap or proprietary map data were used. The map was created by the authors in ArcGIS Pro.

This study focuses on five major metropolitan areas in Saudi Arabia: Riyadh, Makkah, Jeddah, Madinah, and Dammam (Fig 1). These cities represent the kingdom’s most significant urban centers, collectively housing over 20 million inhabitants and exemplifying diverse urbanization patterns across different geographic and economic contexts. Riyadh, the national capital located in the central Najd plateau, serves as the political and administrative center with an estimated population of 7 million inhabitants. The city has experienced unprecedented expansion from a compact desert settlement to a sprawling metropolitan area covering over 1,900 km² [17]. Jeddah, positioned along the Red Sea coast, functions as the kingdom’s principal commercial hub and gateway for Hajj pilgrimage, hosting over 4 million residents. Its coastal location presents unique urbanization dynamics influenced by maritime accessibility and topographic constraints [18]. Makkah, the holiest city in Islam, experiences distinctive urban pressures driven by religious tourism, accommodating over 3 million annual pilgrims in addition to its 2 million permanent residents [19]. The city’s development is significantly constrained by mountainous topography and religious site preservation requirements. Madinah, the second holiest city, is located in the Al-Hijaz region at coordinates 24°28’06"N, 39°36’06"E, surrounded by volcanic hills and spanning approximately 99 km² within its municipal boundaries [20]. Dammam, the capital of the Eastern Province, represents the heart of Saudi Arabia’s petroleum industry with a diverse population of 1.1 million residents, including significant expatriate communities from across the Middle East and Asia [18]. The study areas exhibit diverse topographic characteristics influencing urban development patterns. Riyadh occupies a relatively flat plateau with elevations ranging from 600–800 meters above sea level, while Jeddah’s coastal plain transitions to elevated terrain in the eastern sections. Makkah and Madinah are characterized by mountainous terrain and volcanic landscapes, creating significant topographic constraints for urban expansion. Dammam features relatively flat coastal topography with gentle undulations toward the interior.

### 2.2. Data acquisition and pre-processing

This research utilized 12 Landsat satellite images covering the five study cities, acquired from the United States Geological Survey (USGS) Earth Explorer platform (https://earthexplorer.usgs.gov/) (Table 1). The dataset comprises Landsat 5 Thematic Mapper (TM) imagery from 1993 and Landsat 8 Operational Land Imager (OLI) imagery from 2023, providing a 30-year temporal baseline for change detection analysis.

**Table 1:** Landsat satellite imagery dataset specifications for multi-temporal urban change detection across five Saudi Arabian cities, including sensor types, acquisition dates, cloud cover percentages, and geographic coverage areas (1993-2023).

N0	Path	Row	Sensor	Date	Cloud cover (%)	Covered Area
1	165	42	TM	12 March 1993	6	Dammam
2			OL	5 April 2023	3	
3	166	43	TM	11 May 1993	4	
4			OL	5 June 2023	5	
5	167	43	TM	7 March 1993	0	Riyadh
6			OL	13 April 2023	0	
7	169	44	TM	3 January 1993	0	
8			OL	2 May 2023	0	
9	170	44	TM	12 July 1993	4	Makkah & Madinah
10			OL	30 June 2023	6	
11	171	45	TM	9 August 1993	7	Jeddah
12			OL	17 July 2023	0	

All images were Level 1 terrain-corrected products, geometrically rectified to their corresponding Universal Transverse Mercator (UTM) zones: Zone 37 for Makkah, Jeddah, and Madinah; Zone 38 for Riyadh; and Zone 39 for Dammam [2]. Image selection criteria prioritized cloud-free conditions (≤7% cloud cover) and temporal consistency within seasonal windows to minimize phenological variations. The acquisition dates were strategically selected to avoid extreme seasonal variations, with most images captured during winter and spring months (January-May) to ensure optimal atmospheric conditions and vegetation stability.

Geometric correction was required for two images: the Dammam TM image (Path 154, Row 52) and Riyadh TM image (Path 175, Row 53), which exhibited apparent displacement. These images were geo-referenced using corresponding OLI imagery from identical path-row combinations, employing at least 20 ground control points and achieving root-mean-square errors below 0.5 pixels [15]. Mosaic techniques combined images from adjacent paths and rows for Riyadh and Dammam to ensure complete metropolitan area coverage.

A critical consideration in multi-temporal analysis involved addressing radiometric differences between Landsat TM and OLI sensors. TM imagery operates with 7-bit radiometric resolution (0–127 digital number range), while OLI provides 12-bit resolution (0–4095 range), creating substantial dynamic range disparities [7,8]. To address this issue, digital numbers (DN) were converted to Top of Atmosphere (TOA) radiance using sensor-specific calibration coefficients following the methodology established by Chander et al. [21]:


$$\begin{array}{c} L_{\lambda }={M_{\text{L}}Q}_{\text{CAL}}+\;A_{\text{L}}\; \end{array}$$ (1)

where L_λ_ represents TOA spectral radiance (Watts m^-2^ srad^-1^ μm^-1^), ML and AL are multiplicative and additive rescaling factors from metadata files, and QCAL represents the quantized calibrated pixel value.

### 2.3. Spectral index calculation

To enhance land-cover discrimination and improve classification accuracy in arid environments, three key spectral indices were computed for each image (Table 2). NDVI was computed to identify vegetated areas and distinguish them from urban surfaces [9,10]. SAVI was employed to minimize soil background effects, particularly in sparse-vegetation environments typical of arid regions [22]. In SAVI calculation, L represents the soil brightness correction factor (L = 0.5 for moderate vegetation coverage). NDWI facilitated water body identification and enhanced urban-water boundary delineation [22].

**Table 2:** Spectral indices used for land cover discrimination.

Indices name and equations	References
$𝐍𝐃𝐕𝐈=\frac{{DN}_{𝐍𝐈𝐑}\;-\;{DN}_{𝐑𝐄𝐃}\;}{{DN}_{𝐍𝐈𝐑}+{DN}_{𝐑𝐄𝐃}}$	[9,10]
$𝐒𝐀𝐕𝐈=\frac{{DN}_{𝐍𝐈𝐑}\;-\;{DN}_{𝐑𝐄𝐃}\;}{{DN}_{𝐍𝐈𝐑}+{DN}_{𝐑𝐄𝐃}+L}*(1+L)$	[22]
$𝐍𝐃𝐖𝐈=\frac{{DN}_{𝐍𝐈𝐑}\;-\;{DN}_{SWIR1}\;}{{DN}_{𝐍𝐈𝐑}+{DN}_{SWIR1}}$	[22].

### 2.4. Image classification and accuracy assessment

Urban land cover classification employed a decision tree approach using hierarchical thresholding based on spectral band values and derived indices [23]. Four primary land cover classes were identified: water bodies, vegetation, bare land, and urban areas. The classification hierarchy prioritized classes with highest spectral separability (water and vegetation) before addressing spectrally similar classes (bare land and urban areas). Urban areas were defined as regions dominated by impervious surfaces, including buildings, roads, and other constructed features [24]. This definition follows established urban mapping protocols while accounting for the unique characteristics of desert urban environments where built surfaces may exhibit spectral similarity to natural desert pavements. City-specific classification thresholds were developed to accommodate local environmental conditions and spectral variations (Table 3). For example, Near-Infrared (NIR) responses were utilized for vegetation detection across all cities but extended to sandy desert identification in Riyadh, Jeddah, and Dammam, where extensive sand coverage required specialized handling. Conversely, in Makkah and Madinah, characterized by volcanic and mountainous terrain, NIR applications were limited to vegetation mapping to avoid misclassifying dark volcanic surfaces. Spectral threshold values were derived through an iterative process of visual interpretation and spectral signature sampling from known land cover training areas, informed by published threshold ranges for arid environments [2,23]. For each land cover class, spectral signatures were extracted from representative training pixels across all five cities, and threshold boundaries were refined through repeated classification trials until optimal class separation was achieved. A hierarchical decision-tree approach was adopted, in which spectrally distinct classes (water bodies, followed by vegetation) were classified first and masked from subsequent classification stages. This sequential masking reduced spectral confusion between the remaining classes. The separation of urban and bare land, a well-documented challenge in arid environments due to high spectral overlap between impervious surfaces and exposed desert materials, was addressed by combining SAVI, NDWI, and visible-band thresholds (Table 3). Urban surfaces were distinguished from bare soil primarily by their lower blue-band reflectance (Blue 45–95 DN versus >130 DN for bright sand), slightly negative SAVI values, and characteristic NDWI range. While this multi-criteria approach improved discrimination, residual confusion between these classes is acknowledged as a limitation, particularly in peri-urban transition zones.

**Table 3:** Spectral threshold values used for decision-tree land cover classification.

Class	Feature set	Threshold value
Water	SWIR2	SWIR2 < 25
	SAVI	SAVI < −0.08
Vegetation	NDVI	NDVI > 0.2
	NIR	NIR > 70 and NIR < 110
Bare Soil	NIR	NIR > 120
	BLUE	Blue > 130 and Blue < 150
	RED	Red > 90
	NDVI	NDVI<0
Urban	BLUE	Blue > 45 and Blue < 95
	RED	Red > 55 and Red < 80
	SAVI	SAVI > -0.07 and SAVI < −0.05
	NDWI	NDWI>0.125 and NDWI <0.25

Accuracy assessment followed established RS protocols using stratified random sampling based on land cover class distribution [25]. For 2023 classifications, 450 validation points were generated for each city. Sample size was determined based on minimum statistical requirements for four-class accuracy assessment rather than proportional to city area, following the recommendation of at least 50 samples per class [25]. The stratified approach allocated sample points proportional to class area coverage while ensuring minimum representation for rare classes such as water bodies. Historical accuracy assessment for 1993 imagery presented unique challenges due to limited reference data availability. Following methodological guidance from Foody [26], NDVI change analysis identified pixels with minimal vegetation change between 1993 and 2023, creating a stable pixel population for accuracy assessment. This approach yielded 400 validation samples for larger cities and 330 for Makkah and Madinah, providing robust accuracy estimates while acknowledging inherent limitations in historical validation. Classification accuracy was evaluated using producer’s accuracy, user’s accuracy, overall accuracy, and Cohen’s Kappa coefficient calculated from confusion matrices [25].

### 2.5. Change detection analysis

Post-classification comparison methodology quantified urban expansion by identifying pixel-level transitions between 1993 and 2023 classifications [12]. This approach provides detailed change matrices and enables calculation of both gross and net change statistics for each land cover class. Urban expansion was measured in both absolute terms (km²) and relative terms (percentage change) to facilitate inter-city comparisons despite varying initial urban extents.

Spatial characterization of urban expansion patterns was conducted through visual interpretation of classified maps, supplemented by quantitative area-based change statistics. Urban growth morphology was characterized qualitatively across cities by examining directional expansion trends, development density patterns, and the relationship between urban form and topographic constraints.

### 2.6. Topographic analysis and statistical modeling

Elevation and slope data were derived from the Shuttle Radar Topography Mission (SRTM) 30-meter digital elevation model to analyze topographic influences on urban development patterns. Slope values were calculated using standard GIS algorithms and classified into categories appropriate for urban development assessment.

Binary logistic regression modeling quantified relationships between topographic variables and urban development probability following methodological approaches established by Alqurashi et al. [14]. The model specification included elevation and slope as primary explanatory variables:


$$\begin{array}{c} P(\text{Urban })=\frac{\text{1}}{\text{1}+e^{-\left(\beta_{\text{0}}+\beta_{\text{1}}\cdot \text{ Elevation }+\beta_{\text{2}}\cdot \text{ Slope}\right)}} \end{array}$$

where P(Urban) represents the probability of urban development, β_0_ is the intercept term, and β_1_, β_2_ are regression coefficients for elevation and slope variables, respectively.

Model training employed stratified random sampling with urban areas coded as 1 and non-urban areas as 0. Sample sizes varied by city based on metropolitan extent: 28,000 samples for Riyadh, 18,000 for Jeddah, 22,000 for Dammam, and 9,000 each for Makkah and Madinah. Model performance was evaluated using statistical significance tests, with α = 0.05 as the significance threshold. Model performance was further assessed using Nagelkerke pseudo-R² as a measure of goodness-of-fit, and Receiver Operating Characteristic (ROC) Area Under the Curve (AUC) as a measure of discriminative ability. It should be noted that the model was intentionally restricted to topographic variables (elevation and slope) to isolate the influence of physical terrain characteristics on urban development patterns. Socioeconomic factors including proximity to roads, population density, and economic activity are recognized as important drivers of urban expansion but were beyond the scope of this topography-focused analysis. This analytical scope is acknowledged as a limitation, and the modest explanatory power of topography-only models is expected.

### 2.7. Quality control and validation

Comprehensive quality control procedures ensured data consistency and analytical reliability. Quality control involved three procedures. First, classified images were visually compared against Google Earth high-resolution imagery to identify obvious misclassifications, particularly along urban-desert boundaries where spectral overlap is highest. Second, independent verification using high-resolution imagery was conducted for areas with uncertain classifications to confirm land cover assignments. Third, temporal plausibility screening examined the change matrices for physically impossible transitions (e.g., urban-to-water conversion in inland areas), and pixels flagged as implausible were individually reviewed and corrected where misclassification was confirmed. These procedures are predominantly qualitative in nature and introduce an element of subjectivity, which is acknowledged as a limitation of the current methodology.

**Fig 2:**
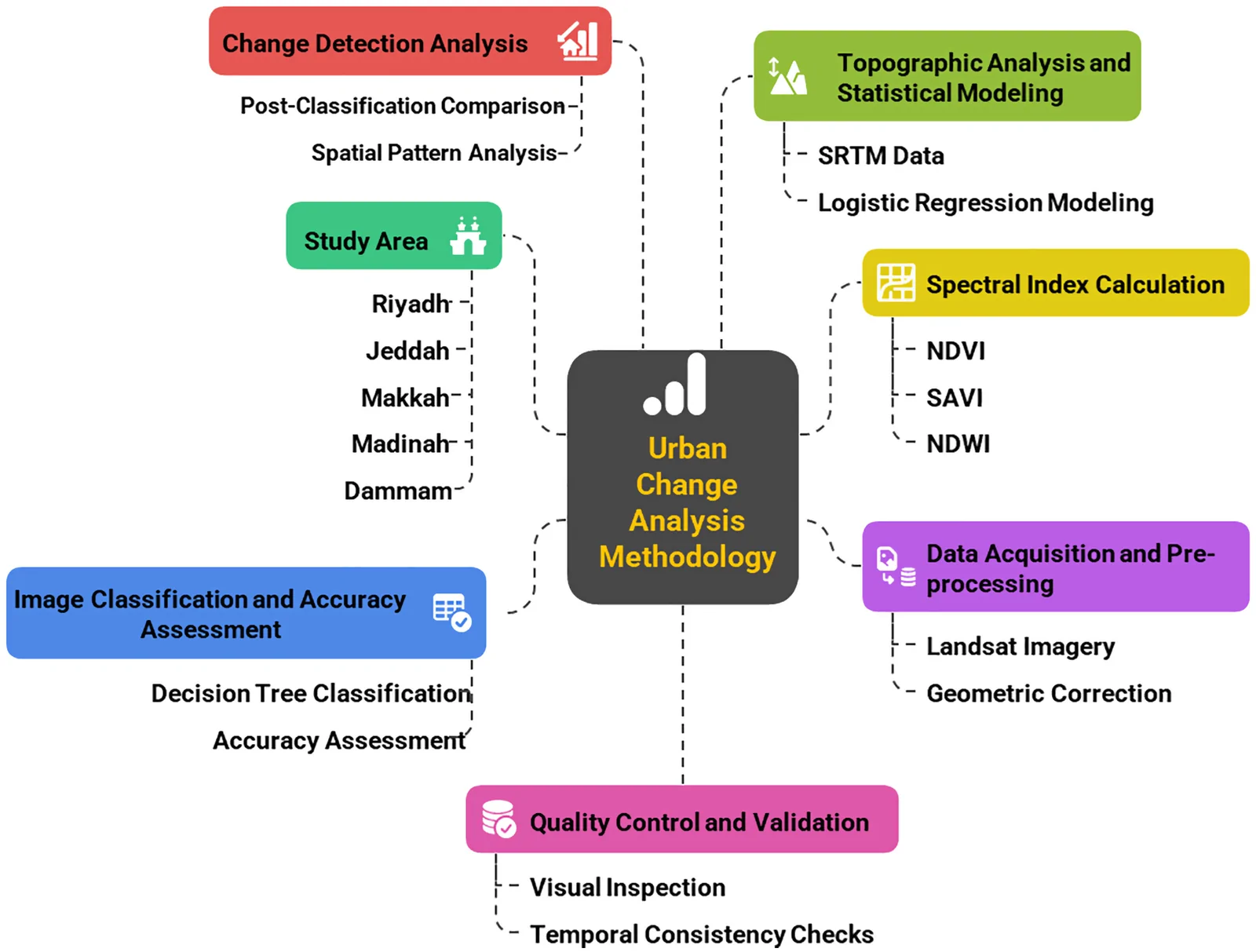
Methodological workflow.

The overall analytical workflow integrated multiple data sources and processing steps to achieve comprehensive urban change analysis (Fig 2). The methodology encompassed data acquisition, preprocessing, classification, change detection, and statistical modeling phases, each with specific quality control procedures. This systematic approach ensured reproducible results while maintaining analytical rigor throughout the multi-temporal analysis process.

## 3. Results

### 3.1. Classification accuracy assessment

The supervised classification of Landsat imagery achieved consistently high accuracy levels across all five study cities and temporal periods. Overall classification accuracies ranged from 92.0% to 95.7%, with Kappa coefficients between 0.874 and 0.937, indicating substantial to excellent agreement between classified results and reference data (Table 4). Riyadh demonstrated the highest classification performance with overall accuracies of 95.2% (1993) and 93.4% (2023), while Dammam showed the most consistent performance across both temporal periods (92.3% and 92.0%, respectively).

**Table 4:** Classification accuracy assessment for all cities and temporal periods.

		User accuracy (%)				Producer accuracy (%)					
Year	City	Water body	Vegetation	Barren land	Built-up	Water body	Vegetation	Barren land	Built-up	Overall accuracy	Kappa statistics
1993	Dammam	0.897	0.943	0.897	0.897	0.875	0.928	0.988	0.78	0.923	0.883
2023		1.00	0.86	0.94	0.91	0.88	0.89	0.95	0.90	0.92	0.874
1993	Jeddah	0.997	0.83	0.97	0.89	1.00	0.83	0.98	0.85	0.942	0.897
2023		1.00	1.00	0.94	0.86	0.82	0.84	0.94	0.95	0.92	0.876
1993	Madinah	0.88	0.98	0.96	0.82	0.88	0.98	0.96	0.80	0.942	0.881
2023		1.00	1.00	0.94	0.90	0.87	0.95	1.00	0.92	0.957	0.937
1993	Makkah	0.982	0.94	0.96	0.87	1.00	0.97	0.95	0.87	0.945	0.900
2023		1.00	1.00	0.90	0.93	1.00	0.83	0.97	0.88	0.92	0.876
1993	Riyadh	1.00	1.00	0.92	1.00	1.00	0.997	0.993	0.85	0.952	0.926
2023		1.00	0.98	0.88	0.98	1.00	0.98	0.99	0.85	0.934	0.901

Producer’s accuracy for urban areas, representing the percentage of reference urban pixels correctly classified, exceeded 85% for all cities and periods, with several instances achieving perfect classification (100%). User’s accuracy for urban classes, indicating the reliability of urban predictions, ranged from 78% to 98%, demonstrating robust classification performance despite the spectral complexity of arid urban environments. Water body classification achieved exceptional accuracy (>87% producer’s accuracy) across all cities, while vegetation classification showed variable performance depending on local vegetation density and seasonal conditions.

The occurrence of perfect (1.0) precision or recall values, observed primarily for water body classification, reflects the strong spectral separability between water surfaces and all other land cover types in the SWIR bands within arid environments. For other classes, accuracy values consistently fell below 1.0, reflecting the spectral complexity inherent in arid urban classification. The most common classification confusion occurred between the urban and barren land classes, particularly in peri-urban transition zones where new construction occurs on exposed desert surfaces. In these areas, the spectral similarity between bright urban materials (concrete, light-colored rooftops) and surrounding sandy surfaces created classification ambiguity that the hierarchical decision-tree approach could only partially resolve. While training and validation samples were generated through independent stratified random sampling, the spatial proximity of samples within relatively homogeneous spectral environments may contribute to optimistic accuracy estimates, a common challenge in RS validation studies.

Additional classification performance metrics for validation, including precision, recall, and F1-score, are provided in Table 5. In the RS context, precision corresponds to the user’s accuracy and recall to the producer’s accuracy; these metrics are presented in machine-learning notation to facilitate comparison with studies employing automated classification approaches. F1-scores for urban classifications consistently exceeded 0.839, confirming adequate classification performance for change detection analysis.

**Table 5:** Additional classification performance metrics (precision, recall, and F1-score) for all cities and temporal periods.

Year	City	Parameter	Water body	Vegetation	Barren land	Built-up	Accuracy	Macro avg	Weighted avg
1993		Precision	1.000	0.897	0.943	0.897	0.934	0.934	0.934
	Dammam	Recall	0.913	0.929	1.000	0.788	0.934	0.907	0.934
		F1-score	0.955	0.912	0.971	0.839	0.934	0.919	0.933
		Precision	1.000	0.833	0.965	0.895	0.942	0.923	0.942
	Jeddah	Recall	1.000	0.833	0.976	0.850	0.942	0.915	0.942
		F1-score	1.000	0.833	0.971	0.872	0.942	0.919	0.942
		Precision	0.875	0.980	0.956	0.824	0.943	0.909	0.942
	Madinah	Recall	0.875	0.980	0.961	0.800	0.943	0.904	0.943
		F1-score	0.875	0.980	0.958	0.812	0.943	0.906	0.942
		Precision	1.000	0.938	0.961	0.867	0.945	0.941	0.945
	Makkah	Recall	1.000	0.968	0.949	0.867	0.945	0.946	0.945
		F1-score	1.000	0.952	0.955	0.867	0.945	0.943	0.945
		Precision	1.000	1.000	0.900	0.933	0.932	0.958	0.937
	Riyadh	Recall	1.000	0.833	1.000	0.875	0.932	0.927	0.932
		F1-score	1.000	0.909	0.947	0.903	0.932	0.940	0.931
2023		Precision	1.000	0.862	0.939	0.910	0.921	0.928	0.921
	Dammam	Recall	0.875	0.893	0.949	0.897	0.921	0.903	0.921
		F1-score	0.933	0.877	0.944	0.904	0.921	0.915	0.921
		Precision	1.000	1.000	0.937	0.862	0.920	0.950	0.925
	Jeddah	Recall	0.818	0.837	0.937	0.953	0.920	0.886	0.920
		F1-score	0.900	0.911	0.937	0.905	0.920	0.913	0.920
		Precision	1.000	1.000	0.944	0.900	0.958	0.961	0.959
	Madinah	Recall	0.867	0.946	1.000	0.918	0.958	0.933	0.958
		F1-score	0.929	0.972	0.971	0.909	0.958	0.945	0.957
		Precision	1.000	1.000	0.900	0.933	0.932	0.958	0.937
	Makkah	Recall	1.000	0.833	1.000	0.875	0.932	0.927	0.932
		F1-score	1.000	0.909	0.947	0.903	0.932	0.940	0.931
		Precision	1.000	0.978	0.880	0.984	0.939	0.961	0.944
	Riyadh	Recall	1.000	0.978	0.986	0.863	0.939	0.957	0.939
		F1-score	1.000	0.978	0.930	0.920	0.939	0.957	0.939

### 3.2. Urban expansion patterns and dynamics

#### 3.2.1. Quantitative urban growth analysis.

The analysis revealed dramatic urban expansion across all five Saudi cities between 1993 and 2023, with total urban area increases ranging from 48.92 km² to 759.2 km² (Table 6). Madinah experienced the largest absolute expansion (759.2 km²), growing from 1,859.20 km² in 1993–2,618.43 km² in 2023, representing a 40.8% increase. Riyadh demonstrated substantial growth with 648.08 km² of new urban area, expanding from 273.65 km² to 921.73 km², constituting a remarkable 236.9% increase, the second-highest growth rate among studied cities.

**Table 6:** Urban area measurements, expansion statistics, and population-normalized growth for the five study cities (1993 to 2023). Population data from the General Authority for Statistics, Kingdom of Saudi Arabia (1992 and 2022 censuses).

City	Area 1993	Area 2023	Change	Growth%	Pop 1992	Pop 2022	Per capita 1993	Per capita 2023	Ann. Growth	Pop Growth%
Riyadh	273.65	921.73	648.08	236.83	2,776,096	6,924,566	98.57	133.11	21.60	149.44
Jeddah	352.93	993.05	640.12	181.37	2,046,251	3,712,917	172.48	267.46	21.34	81.45
Makkah	274.37	323.29	48.92	17.83	965,697	2,385,509	284.12	135.52	1.63	147.02
Madinah	1,859.20	2,618.43	759.23	40.84	608,295	1,411,599	3056.41	1854.94	25.31	132.06
Dammam	168.09	327.72	159.63	94.97	482,321	1,386,166	348.50	236.42	5.32	187.39

Jeddah exhibited significant urban development with 640.12 km² of expansion, growing from 352.93 km² to 993.05 km², representing a 181.3% increase. This coastal metropolis demonstrated the highest absolute growth rate among the five cities when considering proportional expansion relative to initial urban extent. Dammam, despite its smaller initial size, nearly doubled its urban footprint from 168.09 km² to 327.72 km² (159.63 km² increase, 94.9% growth rate). Makkah showed the most constrained expansion with 48.92 km² of new urban development, growing from 274.37 km² to 323.29 km² (17.8% increase), reflecting significant topographic and religious site preservation constraints.

Population-normalized analysis using census data from 1992 and 2022 (General Authority for Statistics, Kingdom of Saudi Arabia) [27] revealed divergent urbanization intensities across cities (Table 6). Riyadh and Jeddah exhibited sprawling development patterns, with urban area growth (236.8% and 181.4%, respectively) substantially outpacing population growth (149.4% and 81.5%), resulting in increased per-capita urban area. In contrast, Makkah experienced densification: population grew by 147.0% while urban area expanded by only 17.8%, reducing per-capita urban area from 284.1 m²/person in 1993 to 135.5 m²/person in 2023. This densification pattern is consistent with topographic constraints forcing vertical rather than horizontal growth. Dammam similarly showed population growth (187.4%) exceeding spatial expansion (95.0%), indicating higher-density development in the later study period. Madinah’s large per-capita urban area (3,056 m²/person in 1993) suggests that the classified urban extent may encompass peri-urban developed land beyond the compact city core.

#### 3.2.2. Spatial patterns of urban development.

**Fig 3:**
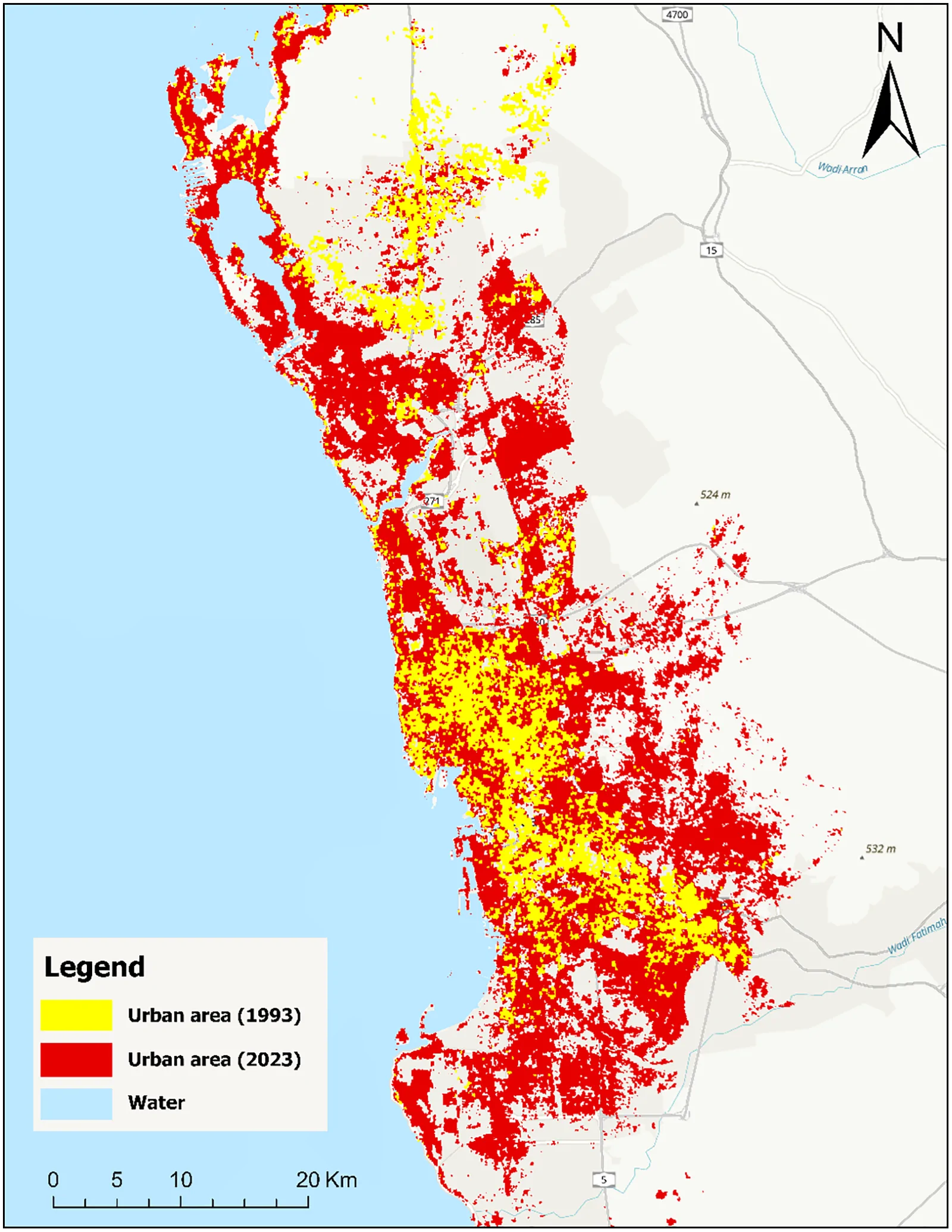
Land cover classification maps for Jeddah (1993 and 2023) showing urban expansion patterns. Derived from Landsat TM and Landsat 8 OLI imagery obtained from USGS Earth Explorer (public domain). Administrative boundaries © NextGIS. No copyrighted basemap or proprietary map data were used. The map was created by the authors in ArcGIS Pro.

Visual analysis of urban expansion maps revealed distinct spatial development patterns across the five metropolitan areas. Jeddah’s urban growth (Fig 3) demonstrated pronounced northward and southward expansion along the Red Sea coastline, with the coastal geography clearly influencing development directionality. The city’s expansion exhibited a linear coastal pattern with secondary growth corridors extending eastward toward elevated inland areas.

**Fig 4:**
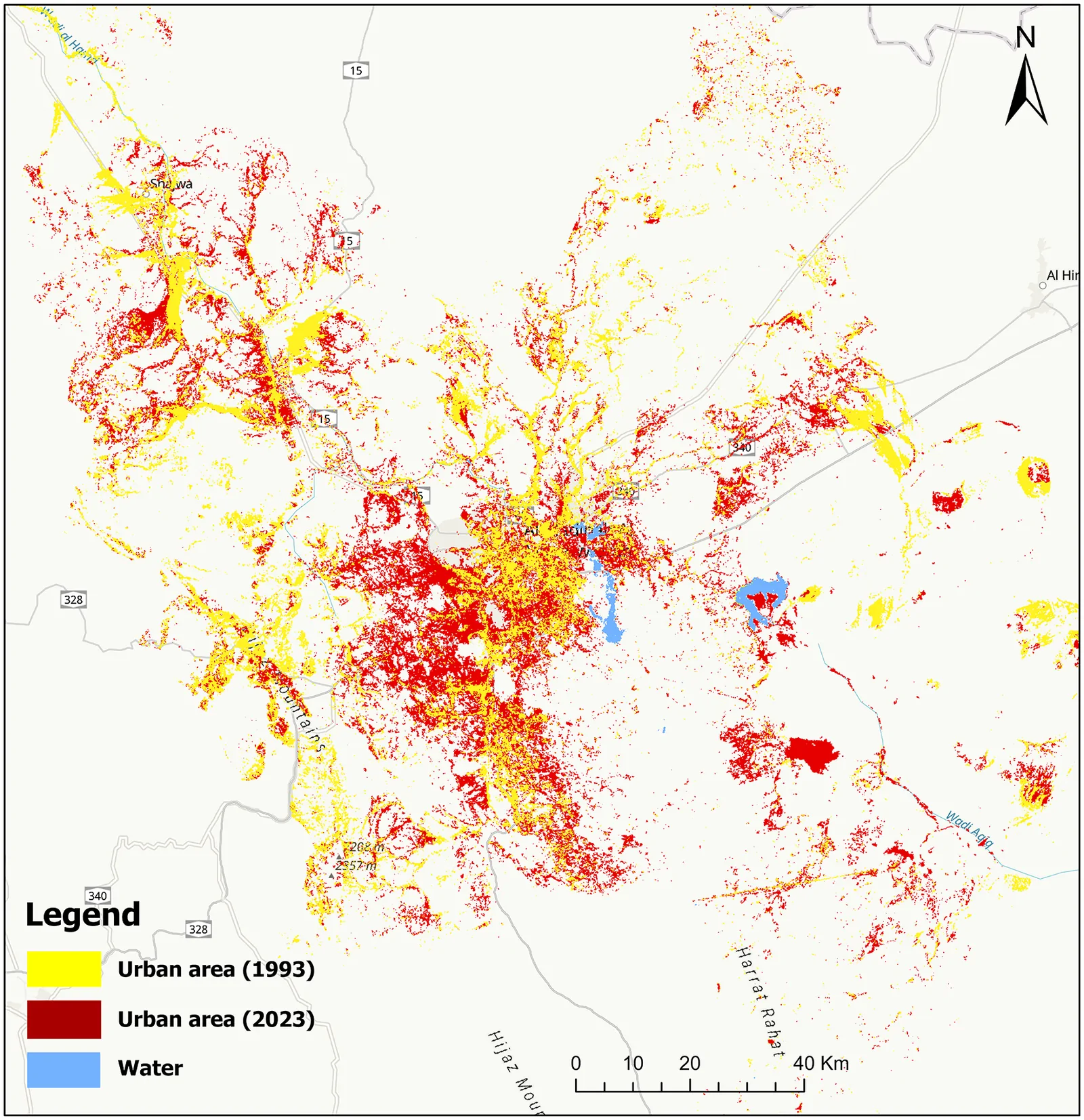
Land cover classification maps for Madinah (1993 and 2023) showing 30-year urban expansion patterns. Derived from Landsat TM and Landsat 8 OLI imagery obtained from USGS Earth Explorer (public domain). Administrative boundaries © NextGIS. No copyrighted basemap or proprietary map data were used. The map was created by the authors in ArcGIS Pro.

Madinah’s urban development (Fig 4) exhibited radial expansion from the historic city center, with growth patterns shaped by the surrounding mountainous terrain. The expansion demonstrated relatively balanced directional growth, though topographic constraints created irregular boundaries and limited development in certain sectors. The city’s unique position among volcanic hills resulted in a distinctive urban morphology adapted to local terrain conditions.

**Fig 5:**
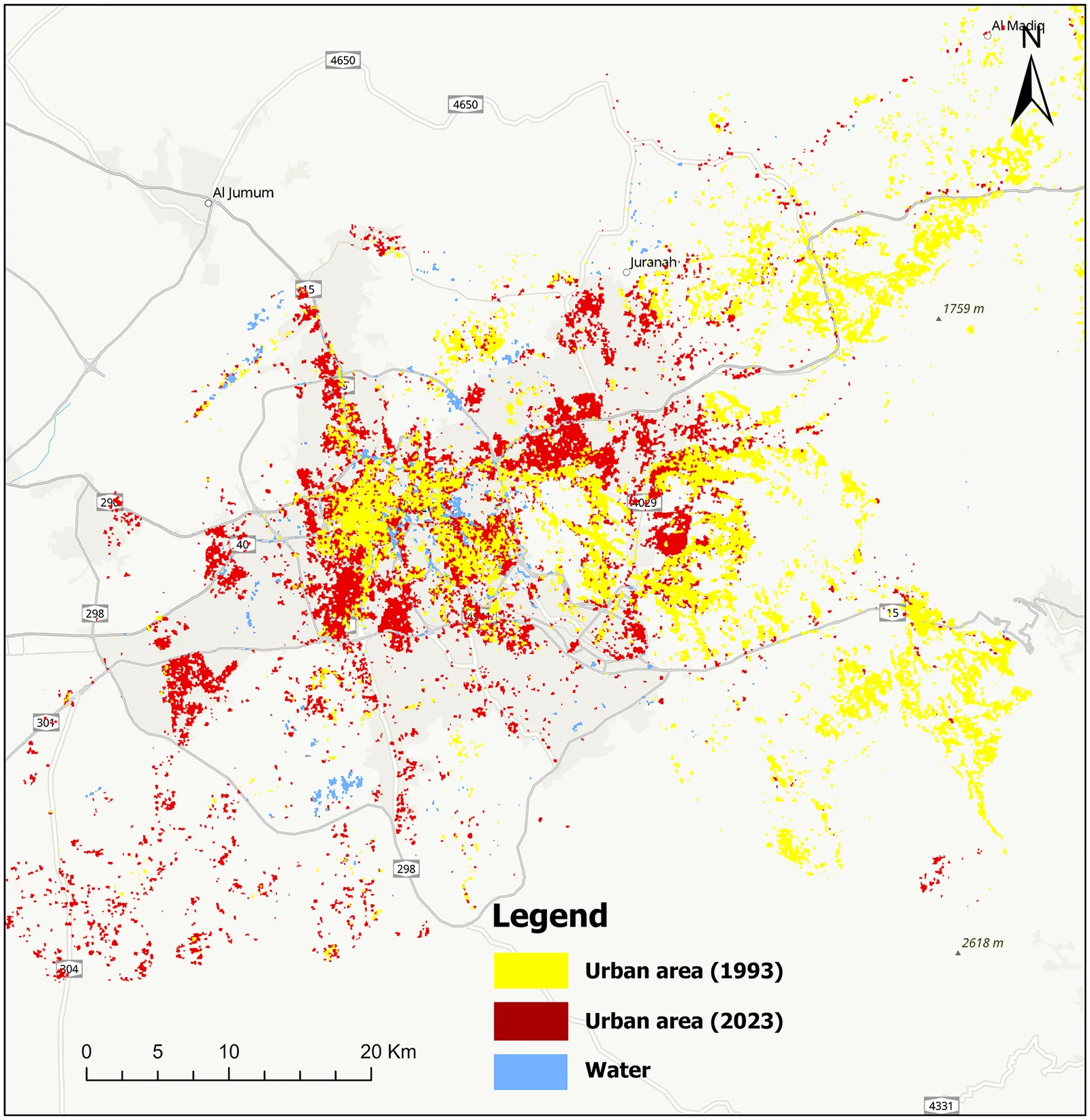
Land cover classification maps for Makkah (1993 and 2023) showing constrained expansion within mountainous terrain. Derived from Landsat TM and Landsat 8 OLI imagery obtained from USGS Earth Explorer (public domain). Administrative boundaries © NextGIS. No copyrighted basemap or proprietary map data were used. The map was created by the authors in ArcGIS Pro.

Makkah’s growth pattern (Fig 5) reflected severe topographic constraints, with urban expansion concentrated in valleys and lower-elevation areas between surrounding mountains. The city’s development showed highly irregular patterns dictated by terrain accessibility and religious site preservation requirements. Despite limited absolute expansion, the spatial analysis revealed intensive development within available suitable areas.

**Fig 6:**
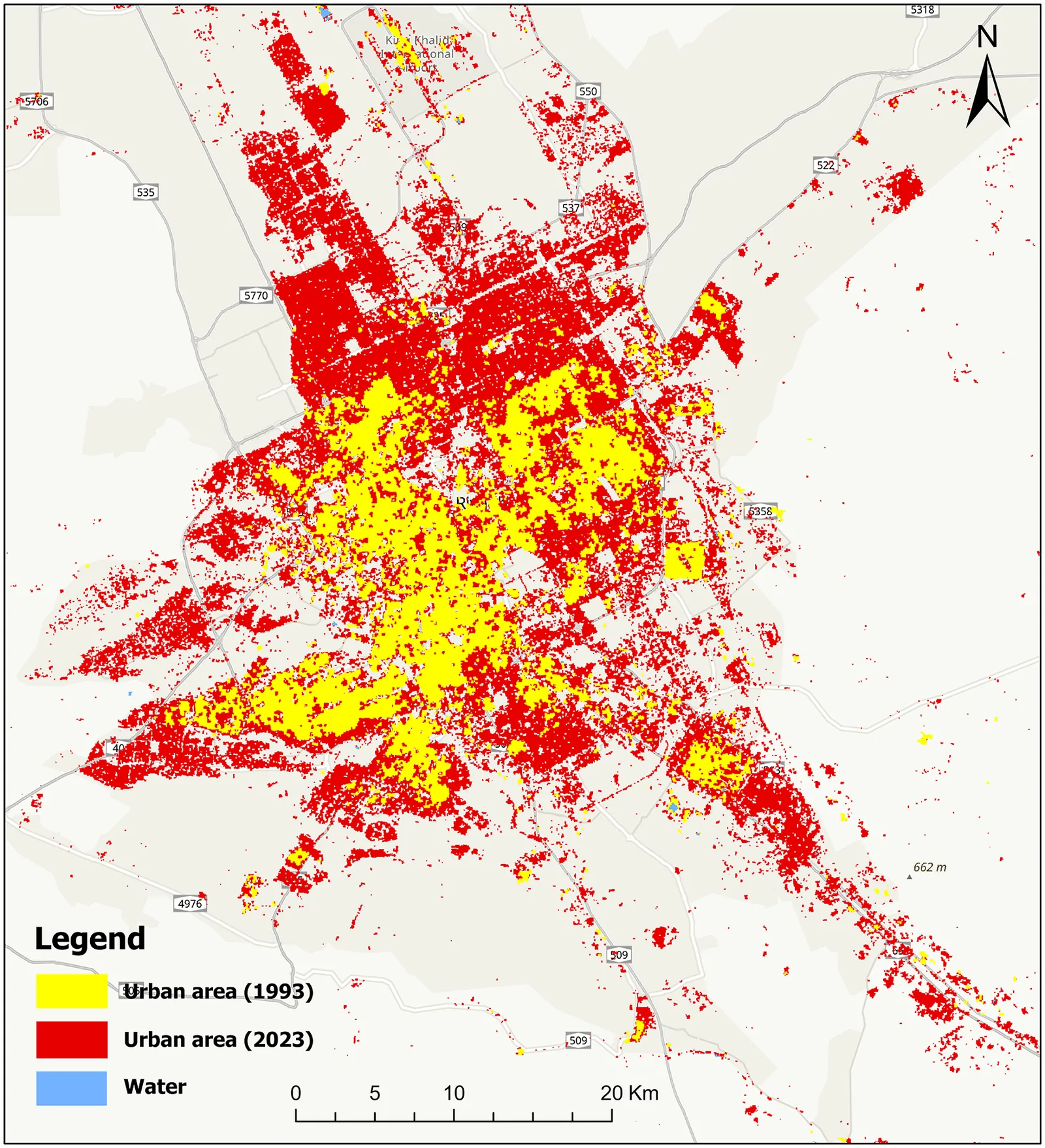
Land cover classification maps for Riyadh (1993 and 2023) showing metropolitan expansion across the Najd plateau. Derived from Landsat TM and Landsat 8 OLI imagery obtained from USGS Earth Explorer (public domain). Administrative boundaries © NextGIS. No copyrighted basemap or proprietary map data were used. The map was created by the authors in ArcGIS Pro.

Riyadh’s urban transformation (Fig 6) exemplified classic sprawling metropolitan expansion, with development radiating outward from the historic city center across the relatively flat Najd plateau. The analysis revealed pronounced expansion toward the north and northeast, with secondary growth nodes developing along major transportation corridors. The city’s expansion pattern included satellite developments in Al-Kharj (75 km south) and Al-Burj (86 km north), indicating regional-scale urban influence.

**Fig 7:**
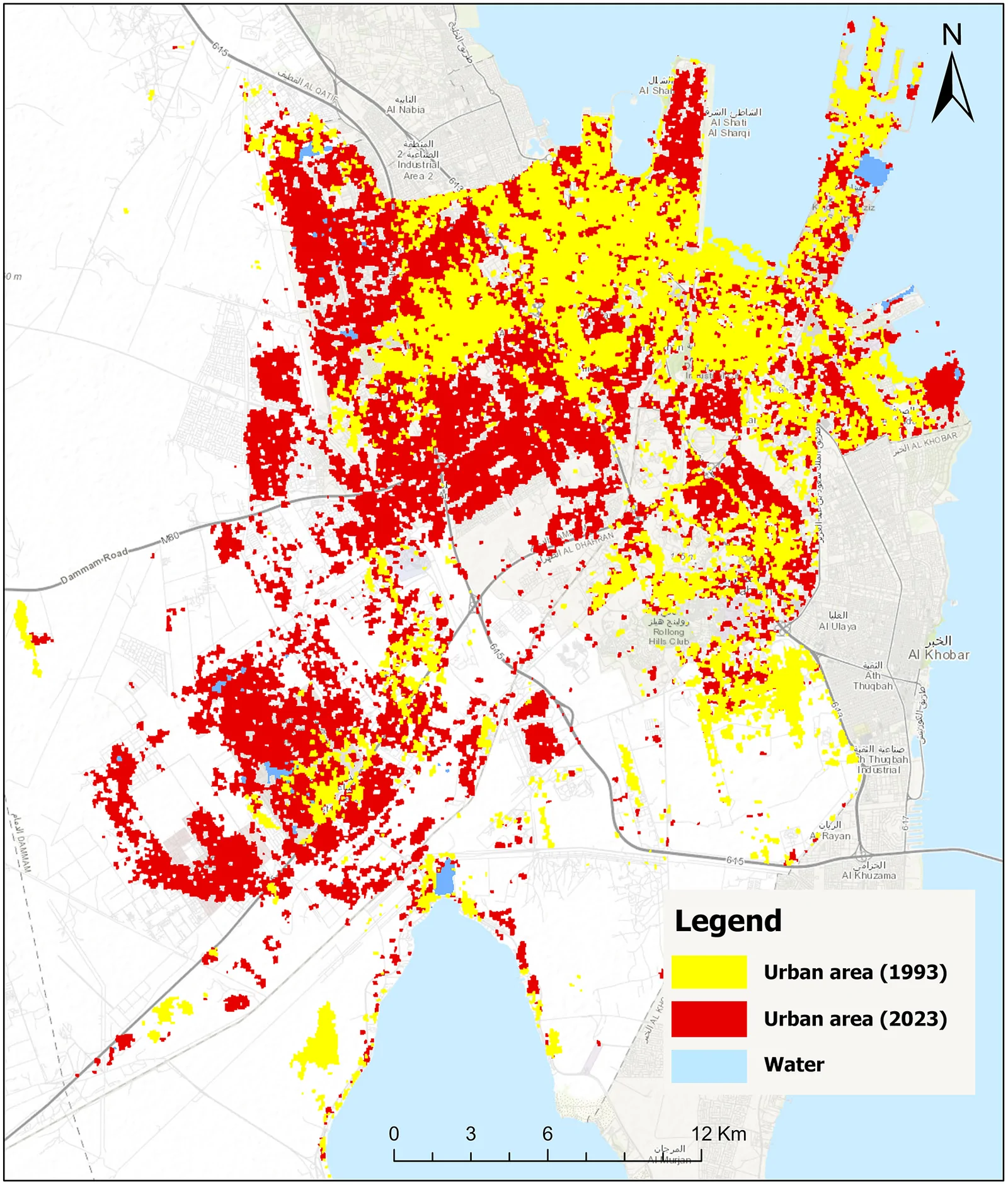
Land cover classification maps for Dammam (1993 and 2023) showing polycentric expansion patterns. Derived from Landsat TM and Landsat 8 OLI imagery obtained from USGS Earth Explorer (public domain). Administrative boundaries © NextGIS. No copyrighted basemap or proprietary map data were used. The map was created by the authors in ArcGIS Pro.

Dammam’s development (Fig 7) demonstrated a polycentric expansion pattern, with growth occurring around multiple urban cores throughout the greater metropolitan area. The eastern coastal sections showed limited expansion due to proximity to the Arabian Gulf, while inland areas experienced substantial development associated with petroleum industry infrastructure.

The three-decade analysis period revealed non-uniform growth rates across different temporal phases, with most cities experiencing accelerated expansion during the latter portion of the study period. This acceleration is consistent with documented increases in oil revenues, government infrastructure investments, and population growth driven by both domestic migration and international immigration [4]. Qualitative assessment of urban morphology indicated that Makkah and Madinah exhibited more compact, centralized development patterns, while Riyadh, Jeddah, and Dammam demonstrated dispersed, sprawling characteristics. This distinction reflects differences in topographic constraints, planning policies, and functional urban roles within the national urban hierarchy.

### 3.3. Topographic influences on urban development

#### 3.3.1. Elevation and slope distribution analysis.

**Fig 8:**
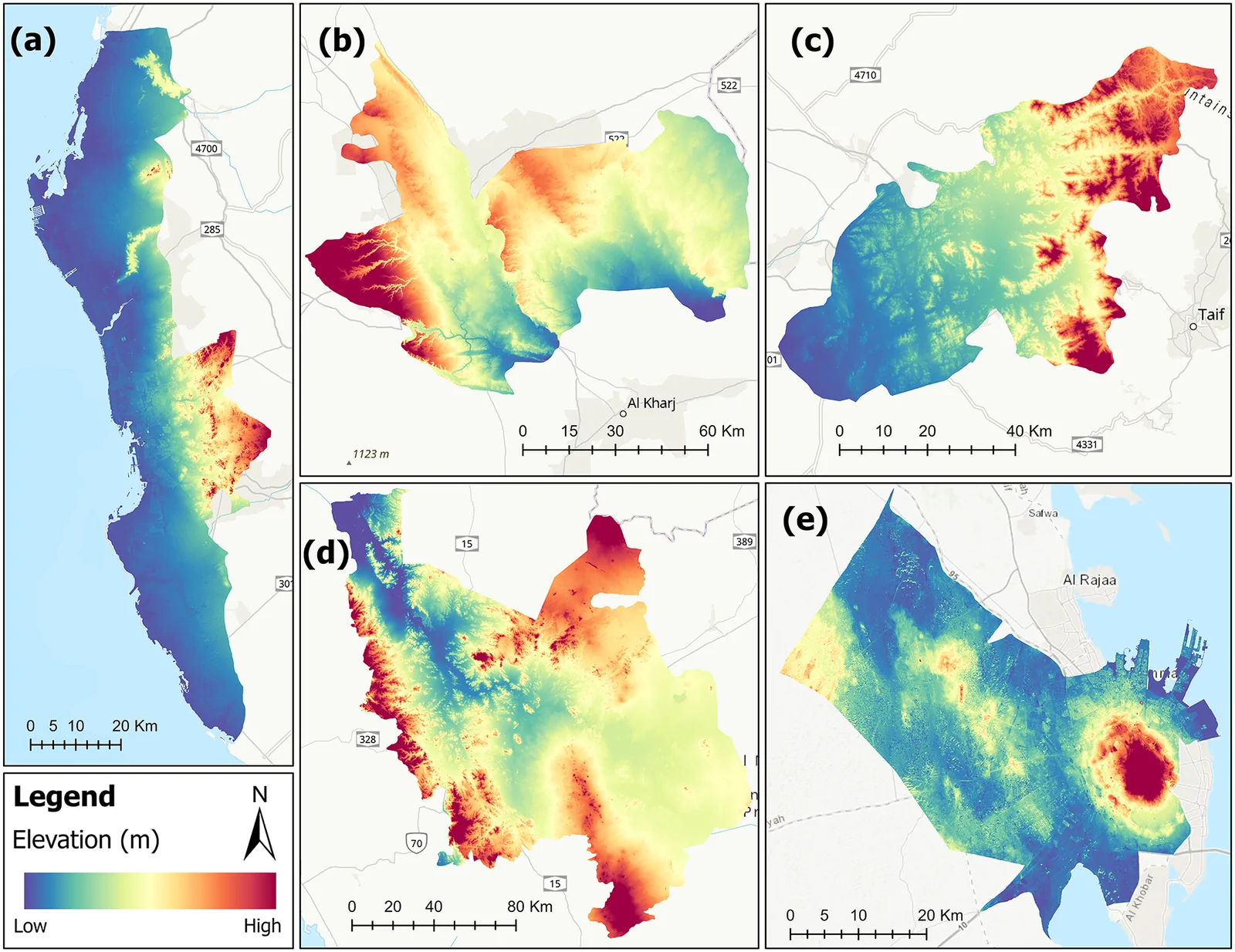
Elevation maps for the five study cities: (a) Jeddah, (b) Riyadh, (c) Makkah, (d) Madinah, and (e) Dammam. Elevation in meters above sea level. Derived from NASA SRTM 30-meter digital elevation model (public domain). Administrative boundaries © NextGIS. No copyrighted basemap or proprietary map data were used. The map was created by the authors in ArcGIS Pro.

**Fig 9:**
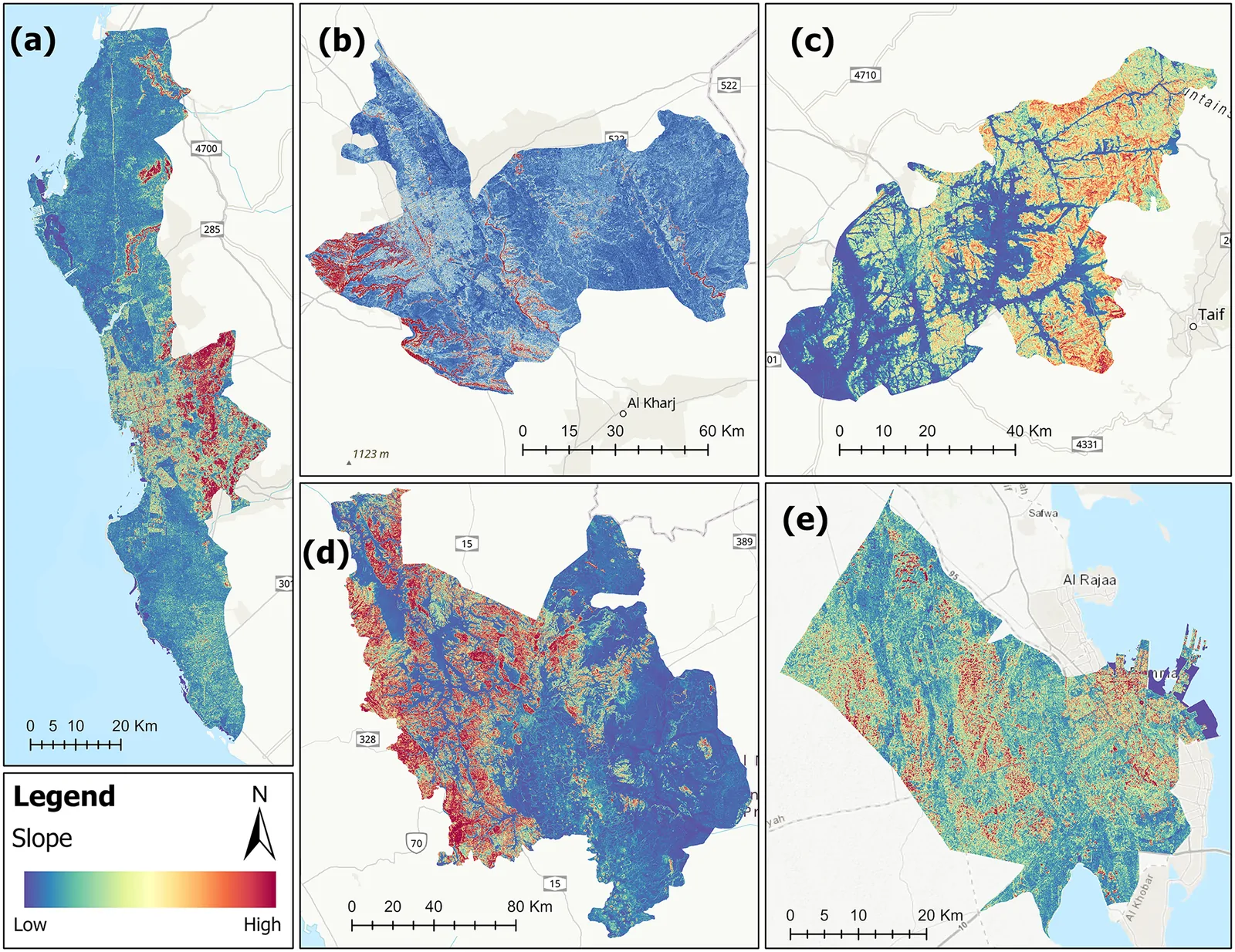
Slope maps for the five study cities: (a) Jeddah, (b) Riyadh, (c) Makkah, (d) Madinah, and (e) Dammam. Slope in percentage. Derived from NASA SRTM 30-meter digital elevation model (public domain). Administrative boundaries © NextGIS. No copyrighted basemap or proprietary map data were used. The map was created by the authors in ArcGIS Pro.

Spatial analysis of topographic characteristics revealed significant variations in elevation and slope conditions across the five study cities (Figs 8 and 9). Jeddah exhibited the most diverse elevation profile (Fig 8a), with coastal areas near sea level transitioning to elevated terrain exceeding 800 meters in eastern sections. This elevation gradient created distinct zones of development suitability, with coastal plains experiencing intensive urbanization while elevated areas remained largely undeveloped. Riyadh’s topography (Fig 8b) showed relatively uniform elevation across the central plateau region (600–800 meters), with gentle undulations that posed minimal constraints to urban expansion. This topographic homogeneity contributed to the city’s extensive sprawling development pattern observed in the spatial analysis. Makkah’s mountainous setting (Fig 8c) created a complex topographic environment with elevation variations exceeding 1,000 meters within the metropolitan area. The surrounding mountains formed natural barriers that channeled urban development into specific valleys and basins, explaining the constrained expansion patterns observed in the land cover analysis. Madinah’s volcanic landscape (Fig 8d) posed unique topographic challenges, with scattered volcanic cones and lava fields resulting in an irregular elevation profile. These features influenced development patterns by creating both obstacles and opportunities for urban expansion. Dammam’s coastal topography (Fig 8e) showed minimal elevation variation, with gentle slopes from inland areas toward the Arabian Gulf. This relatively flat terrain facilitated unrestricted urban expansion in most directions except eastward toward the coast.

Slope analysis (Fig 9) revealed corresponding patterns of terrain ruggedness across the five cities. Makkah and Madinah exhibited the steepest terrain, with extensive areas exceeding 15% slope that significantly constrain urban development. Jeddah exhibited moderate slope conditions in the eastern elevated areas, while Riyadh and Dammam showed predominantly gentle slopes suitable for urban development.

#### 3.3.2. Statistical analysis of topographic controls.

Binary logistic regression analysis quantified the statistical relationships between topographic variables and the probability of urban development across the five cities (Table 7). The results revealed significant topographic influences on urbanization patterns, though the specific relationships varied considerably among cities due to local geographic conditions.

**Table 7:** Binary logistic regression results for topographic influences on urban development probability across the five study cities.

City	Variable	Coefficient	Std. Error	z-Statistic	p-value	Odds Ratio	Nagelkerke R²	ROC AUC
Dammam	Intercept	−0.831	0.188	−4.379	0.0000	–	0.06	0.56
	Elevation	0.0015	0.0041	0.365	0.714	1.0015		
	Slope	−0.0187	0.0239	−0.782	0.434	0.982		
Riyadh	Intercept	−4.860	1.282	−3.788	0.0002	–	0.14	0.64
	Elevation	0.0068	0.0021	3.238	0.0012	1.0068		
	Slope	−0.0089	0.0402	−0.223	0.825	0.993		
Jeddah	Intercept	−1.165	0.296	−3.946	0.0001	–	0.24	0.72
	Elevation	−0.0034	0.0013	−2.616	0.0089	0.997		
	Slope	−0.0466	0.0158	−2.930	0.0034	0.954		
Makkah	Intercept	−0.284	0.379	−0.718	0.473	–	0.19	0.68
	Elevation	−0.0031	0.0014	−2.214	0.0268	0.996		
	Slope	−0.038	0.0174	−2.172	0.0297	0.962		
Madinah	Intercept	0.534	0.682	0.777	0.438	–	0.15	0.66
	Elevation	−0.0032	0.0012	−2.666	0.0077	0.996		
	Slope	−0.0241	0.0128	−1.8828	0.0597	0.9762		

Slope emerged as a statistically significant factor in determining the probability of urban development in Jeddah (β = −0.047, p = 0.003) and Makkah (β = −0.038, p = 0.030), with a marginally significant effect in Madinah (β = −0.024, p = 0.060). In contrast, slope was not statistically significant in Riyadh (p = 0.825) or Dammam (p = 0.434), consistent with the limited topographic variation in these relatively flat urban environments. The negative slope coefficients in Jeddah and Makkah confirm that steeper terrain inhibits urban development, with each additional degree of slope reducing the odds of urbanization by approximately 4.5% in Jeddah (odds ratio = 0.954) and 3.8% in Makkah (odds ratio = 0.962).

Elevation significantly influenced urban development patterns in four of the five cities. Riyadh exhibited a positive elevation coefficient (β = 0.0068, p = 0.001), indicating a preference for slightly elevated locations within the plateau environment. Conversely, Jeddah (β = −0.003, p = 0.009), Makkah (β = −0.003, p = 0.027), and Madinah (β = −0.003, p = 0.008) showed negative elevation effects, confirming that urban development in these cities concentrates at lower elevations, in valleys, coastal plains, and basins rather than on elevated terrain. Dammam’s minimal elevation range produced no significant elevation effect (p = 0.714).

Model fit statistics indicate that topographic variables alone provide modest explanatory power, with Nagelkerke R² values ranging from 0.06 (Dammam) to 0.24 (Jeddah) and ROC AUC values between 0.56 and 0.72 (Table 7). These results confirm that while topographic constraints meaningfully shape urban development patterns, particularly in cities with pronounced terrain variation, socioeconomic and infrastructure variables would be necessary to more fully model urban growth processes.

The pattern of topographic influence corresponds clearly to the physical geography of each city. Jeddah, with the strongest coastal-to-mountain gradient, showed the highest model fit (R² = 0.24, AUC = 0.72) and significant effects for both variables. Makkah’s mountainous setting produced significant effects for both elevation and slope. Madinah’s scattered volcanic terrain yielded a significant elevation effect but only marginal slope influence, reflecting localized rather than continuous topographic barriers. The flat terrain cities, Riyadh and Dammam, showed weak or absent slope effects, confirming that where topographic variation is minimal, terrain alone cannot predict urbanization patterns.

### 3.4. Environmental implications of urban expansion

The comprehensive spatial analysis revealed significant environmental implications associated with the documented urban expansion patterns. The transformation of 2,255.98 km² of natural desert landscape to urban land use across the five cities represents a substantial alteration of regional ecosystem structure and function. This conversion has likely displaced native desert vegetation communities and created extensive impervious surface coverage that may contribute to increased surface runoff and reduced groundwater recharge, consistent with documented urbanization-hydrology relationships in arid regions.

Urban heat island effects are likely present in the larger metropolitan areas, consistent with established relationships between impervious surface coverage and thermal modification in arid climates. This thermal modification compounds the already extreme temperatures characteristic of the Arabian Peninsula climate, potentially increasing energy consumption for cooling and affecting human comfort and health. However, land surface temperature was not directly measured in this study, and these inferences are based on the spatial extent and pattern of impervious surface expansion rather than thermal data.

The spatial patterns of urban expansion suggest potential fragmentation of remaining natural areas, particularly in the peripheries of rapidly growing cities such as Riyadh and Jeddah. Such fragmentation may reduce habitat connectivity and could threaten the persistence of native species adapted to desert environments, while creating edge effects that can alter ecosystem processes in adjacent natural areas.

## 4. Discussion

### 4.1. Urban expansion dynamics and regional context

The quantitative analysis of urban expansion across five major Saudi cities reveals unprecedented rates of metropolitan growth over the 30-year study period (1993–2023), with total urban area increases ranging from 48.92 km² to 759.2 km². These findings align with broader patterns of rapid urbanization observed in developing economies, particularly those experiencing oil-driven economic growth [28]. The dramatic expansion documented in this study, with cities like Riyadh experiencing 236.9% growth and Jeddah achieving 181.3% expansion, represents some of the highest urban growth rates globally, reflecting the unique combination of resource wealth, government investment, and strategic development policies characterizing Saudi Arabia’s urbanization trajectory. The observed expansion patterns demonstrate clear relationships between urban growth drivers and spatial development outcomes. Madinah’s largest absolute expansion (759.2 km²) reflects its dual role as a religious center and regional administrative hub, supported by extensive government infrastructure investments to accommodate increasing pilgrimage volumes [4]. The city’s growth pattern illustrates how religious tourism infrastructure creates multiplier effects that extend far beyond immediate pilgrimage facilities, generating residential, commercial, and service sector development across the broader metropolitan area.

Riyadh’s transformation from a 273.65 km² compact desert settlement to a 921.73 km² sprawling metropolis exemplifies classic patterns of capital city development in resource-rich nations. The city’s expansion reflects centralized government decision-making, substantial growth in public-sector employment, and strategic positioning as the kingdom’s political and administrative center. This growth pattern parallels observations from other oil-producing nations, in which capital cities experience disproportionate expansion due to the concentration of resource revenue and government-led development initiatives [29].

Jeddah’s coastal urbanization dynamics, with 640.12 km² of expansion along the Red Sea coastline, demonstrate how geographic positioning influences metropolitan development patterns. As the kingdom’s principal commercial gateway and Hajj entry point, Jeddah’s growth reflects both economic diversification and the requirements of religious tourism infrastructure. The city’s linear coastal expansion pattern, clearly evident in Figure 3, illustrates how topographic features channel urban development while creating unique environmental challenges for the preservation of coastal ecosystems.

### 4.2. Topographic constraints and urban development patterns

The statistical analysis of topographic influences provides crucial insights into how environmental factors shape urban expansion in arid regions. The statistical analysis reveals a clear gradient in topographic influence corresponding to terrain complexity. Cities with pronounced topographic variation, Jeddah, with its coastal-to-mountain gradient (slope p = 0.003), and Makkah, with its encircling mountain barriers (slope p = 0.030), demonstrated significant negative slope effects on urbanization probability. In contrast, the flat terrains of Riyadh (slope p = 0.825) and Dammam (slope p = 0.434) showed no significant slope influence, confirming that minimal terrain variation limits the predictive capacity of topographic models. These findings complement spatial analysis results showing how elevation and slope distributions create distinct zones of development suitability across the five metropolitan areas.

Makkah’s constrained expansion (48.92 km², 17.8% growth) directly reflects severe topographic limitations imposed by surrounding mountainous terrain. The city’s development pattern, concentrated in valleys and lower-elevation areas between mountains, demonstrates how religious significance, combined with challenging topography, creates a unique urban morphology. This constrained growth pattern contrasts sharply with the sprawling development observed in topographically unrestricted cities like Riyadh and Dammam, highlighting the fundamental role of physical geography in shaping urbanization outcomes.

Elevation coefficients differed in direction between cities. Riyadh exhibited a positive coefficient (β = 0.0068, p = 0.001), indicating preference for slightly higher locations within the plateau, possibly reflecting drainage advantages. In contrast, Jeddah showed a negative elevation coefficient (β = −0.0034, p = 0.009), indicating that urban development concentrates at lower coastal elevations rather than in the elevated eastern terrain. This pattern is consistent with the accessibility and economic advantages of the coastal plain for commercial and residential development compared to low-lying coastal areas.

The elevation was also significant in Makkah (p = 0.027) and Madinah (p = 0.008), with negative coefficients indicating that development in these cities is concentrated in valleys and lower terrain. Only in Dammam did elevation show no significant effect, reflecting the limited elevation range across its flat coastal landscape. These differential responses across cities underscore the importance of site-specific analysis in understanding urban-environment relationships in arid regions.

### 4.3. Environmental implications and sustainability challenges

The comprehensive transformation of 2,255.93 km² of natural desert landscape to urban land use across the five cities represents a substantial alteration of regional ecosystem structure and function. This conversion magnitude is consistent with the rapid pace of Saudi Arabia’s urbanization process, which has been linked to oil-driven economic growth [15]. The potential environmental implications extend beyond simple land cover conversion to encompass complex ecosystem disruptions, habitat fragmentation, and altered hydrological processes.

Based on the spatial patterns of expansion and consistent with findings by Alberti & Marzluff [30], urban heat island effects, particularly pronounced in extensively paved metropolitan areas like Riyadh and Jeddah, compound already extreme desert temperatures and create substantial energy consumption burdens. The spatial patterns of expansion documented in Figs 6 and 3 show extensive low-density sprawl that maximizes impervious surface area while minimizing vegetation coverage, creating optimal conditions for heat island development. These thermal modifications have cascading effects on energy demand, air quality, and human health, particularly during summer months when ambient temperatures already approach physiological limits [30]. Although land surface temperature was not directly measured in this study, the documented patterns of low-density sprawl are consistent with conditions known to intensify urban heat island effects.

Water resource implications represent perhaps the most critical environmental challenge identified through this spatial analysis. The documented urban expansion patterns increase water demand precisely in regions with limited renewable water resources, necessitating expensive desalination and long-distance water-transfer infrastructure. The sprawling development patterns observed in Riyadh and Jeddah particularly exacerbate water infrastructure requirements by dispersing demand across extensive areas rather than concentrating it in compact, well-serviced urban cores.

Biodiversity impacts, while not directly quantified in this study, are evident from the spatial analysis of urban expansion patterns. The fragmentation of remaining natural areas, particularly visible in the peripheries of rapidly growing cities, may reduce habitat connectivity and could threaten the persistence of native species adapted to desert environments [3]. Edge effects from urban development alter ecosystem processes in adjacent natural areas, potentially compromising the ecological integrity of protected areas and biodiversity conservation zones.

### 4.4. Planning and policy implications

The spatial patterns documented in this study reveal significant opportunities for improved urban planning and environmental management. Comparative analysis across five cities demonstrates that topographic constraints, while limiting development options, can channel growth in ways that minimize environmental impacts when properly managed. Makkah’s constrained yet intensive development model, despite its limitations, achieves higher urban density and potentially greater resource efficiency than the sprawling patterns observed in topographically unrestricted cities.

The government-led development initiatives documented across all five cities, supported by substantial oil revenues and strategic infrastructure investments, create unique opportunities for implementing sustainable planning practices [31]. The extensive public sector involvement in urban development provides mechanisms for environmental protection that may not be available in market-driven development contexts, though this study suggests these mechanisms have not been fully utilized to date.

Transportation infrastructure development, clearly evident in the spatial expansion patterns, particularly around Riyadh and Dammam, creates opportunities for coordinated land use and transit planning. The radial expansion patterns observed in these cities could support efficient public transit systems if development densities were increased along major corridors rather than continued low-density sprawl across the broader metropolitan area.

### 4.5. Global-regional implications and limitations

The urbanization patterns documented in Saudi Arabia provide important insights for other rapidly developing arid regions, particularly in the Middle East and North Africa, where similar combinations of resource wealth, government-led development, and environmental constraints create comparable planning challenges. The spatial analysis methods and topographic modeling approaches developed in this study offer transferable tools for analyzing urban-environment relationships in other arid contexts.

The environmental challenges identified through this analysis, particularly water resource constraints, heat island effects, and ecosystem fragmentation, are increasingly relevant globally as urbanization expands into marginal environments. The Saudi experience provides valuable lessons for managing rapid urban growth while minimizing environmental impacts, though it also demonstrates the challenges of achieving sustainable development in resource-rich but environmentally constrained settings.

Climate change implications of the documented expansion patterns extend beyond local environmental impacts to encompass regional and global significance. The extensive urban development in one of the world’s hottest regions creates substantial cooling energy demands that contribute to greenhouse gas emissions, while replacing natural desert surfaces with built environments alters regional albedo and heat-transfer characteristics.

The binary logistic regression approach for analyzing topographic influences provides a replicable framework for quantifying environmental controls on urban development. However, the analysis is limited by the exclusive focus on elevation and slope variables, while other potentially important factors such as proximity to infrastructure, economic zones, and existing urban areas were not incorporated. Future research should expand the analytical framework to include socioeconomic and infrastructure variables alongside physical geographic factors.

The 30-year temporal span provides valuable insights into long-term urbanization trends but may obscure important sub-decadal variations in growth rates and spatial patterns. Higher temporal-resolution analysis using annual or biannual imagery could reveal how urban expansion responds to specific policy initiatives, economic cycles, and infrastructure investments.

## 5. Conclusion

This study examined spatiotemporal patterns of urban expansion across five major Saudi Arabian cities over a 30-year period (1993–2023) using multi-temporal Landsat imagery, post-classification change detection, and binary logistic regression modeling of topographic controls. The analysis revealed that all five cities experienced substantial urban growth during the study period, though the magnitude and spatial character of expansion differed considerably among cities depending on local terrain conditions, functional urban roles, and geographic setting. Riyadh and Madinah recorded the largest absolute increases in urban areas, while Makkah exhibited the most constrained expansion due to severe topographic limitations imposed by its mountainous surroundings. Population-normalized analysis further revealed that Riyadh and Jeddah followed sprawling development trajectories in which urban area growth outpaced population growth, whereas Makkah and Dammam experienced densification, with population increases exceeding spatial expansion.

The binary logistic regression analysis demonstrated that the influence of topographic variables on the probability of urbanization corresponds directly to terrain complexity. Slope was a significant predictor of urban development in cities with pronounced topographic gradients, particularly Jeddah and Makkah, while it had no significant effect in the relatively flat environments of Riyadh and Dammam. Elevation proved significant in four of the five cities, with negative coefficients in Jeddah, Makkah, and Madinah, confirming that development in these cities is concentrated at lower elevations, in coastal plains, valleys, and basins. Model fit statistics (Nagelkerke R² ranging from 0.06 to 0.24) indicate that topographic variables alone provide modest explanatory power, and the inclusion of socioeconomic factors such as proximity to transportation infrastructure, population density, and economic activity would be necessary for more complete modeling of urban growth dynamics.

The environmental implications of the documented expansion were inferred from observed patterns of land-cover conversion rather than measured directly through environmental indicators. The transformation of natural desert landscapes into impervious urban surfaces across the five cities is consistent with conditions known to intensify urban heat island effects, alter hydrological processes, and fragment remaining natural habitats. However, these environmental outcomes were not directly quantified using indicators such as land surface temperature or vegetation loss indices, and this limitation should be addressed in future research by integrating thermal and ecological datasets with the land cover change analysis presented here.

Methodologically, the study demonstrates the effectiveness of multi-temporal Landsat analysis combined with spectral indices (NDVI, SAVI, and NDWI) for quantifying urban change in arid environments, achieving classification accuracies between 92% and 95% despite the spectral similarity between urban surfaces and desert materials that poses a persistent classification challenge. The hierarchical decision-tree classification approach, while effective in reducing confusion between spectrally distinct classes, could not fully resolve the overlap between urban and barren land classes in peri-urban transition zones. Future studies should explore machine learning classification approaches and object-based image analysis to improve discrimination in these challenging spectral environments.

The findings of this research have practical relevance for urban planning and environmental management in Saudi Arabia, particularly in the context of ongoing development programs under Vision 2030. The comparative analysis across five cities with distinct geographic settings demonstrates that topographic constraints, while limiting development options, can channel growth in ways that promote higher density and potentially greater resource efficiency. Future research should prioritize higher-temporal-resolution analyses to capture sub-decadal growth dynamics, expand modeling frameworks to incorporate socioeconomic and infrastructure variables, and directly quantify environmental impacts using land-surface temperature, vegetation change, and hydrological modeling. The integration of climate projections with urban expansion scenarios could further inform adaptation strategies for managing continued urbanization under increasing environmental stress in arid regions.
