# Supplementary material for: Spatiotemporal analysis of urban expansion and environmental impacts in five Saudi Arabian cities by utilizing geospatial and remote sensing approach
Source: PLoS One. 2026 Sep 1;21(9):e0354154. doi: 10.1371/journal.pone.0354154 (PMC13533391; doi:10.1371/journal.pone.0354154)
Supplement: Peer review history

# Peer review: Decision Letter 0

**DOI:** 10.1371/journal.pone.0354154.r001

**Authors:** Laxmi Kant Sharma

7 Mar 2026

Thank you for submitting your manuscript to PLOS ONE. After careful consideration, we feel that it has merit but does not fully meet PLOS ONE’s publication criteria as it currently stands. Therefore, we invite you to submit a revised version of the manuscript that addresses the points raised during the review process.

Please submit your revised manuscript by Apr 21 2026 11:59PM. If you will need more time than this to complete your revisions, please reply to this message or contact the journal office at plosone@plos.org. When you're ready to submit your revision, log on to https://www.editorialmanager.com/pone/ and select the 'Submissions Needing Revision' folder to locate your manuscript file.

- A letter that responds to each point raised by the academic editor and reviewer(s). You should upload this letter as a separate file labeled 'Response to Reviewers'.
- A marked-up copy of your manuscript that highlights changes made to the original version. You should upload this as a separate file labeled 'Revised Manuscript with Track Changes'.
- An unmarked version of your revised paper without tracked changes. You should upload this as a separate file labeled 'Manuscript'.

If you would like to make changes to your financial disclosure, please include your updated statement in your cover letter. Guidelines for resubmitting your figure files are available below the reviewer comments at the end of this letter.

If applicable, we recommend that you deposit your laboratory protocols in protocols.io to enhance the reproducibility of your results. Protocols.io assigns your protocol its own identifier (DOI) so that it can be cited independently in the future. For instructions see: https://journals.plos.org/plosone/s/submission-guidelines#loc-laboratory-protocols. Additionally, PLOS ONE offers an option for publishing peer-reviewed Lab Protocol articles, which describe protocols hosted on protocols.io. Read more information on sharing protocols at https://plos.org/protocols?utm_medium=editorial-email&utm_source=authorletters&utm_campaign=protocols.

We look forward to receiving your revised manuscript.

Kind regards,

Laxmi Kant Sharma, PhD

Academic Editor

PLOS One

Journal Requirements:

When submitting your revision, we need you to address these additional requirements.

1.Please ensure that your manuscript meets PLOS ONE's style requirements, including those for file naming. The PLOS ONE style templates can be found at

https://journals.plos.org/plosone/s/file?id=wjVg/PLOSOne_formatting_sample_main_body.pdf and

https://journals.plos.org/plosone/s/file?id=ba62/PLOSOne_formatting_sample_title_authors_affiliations.pdf

2. We note that the grant information you provided in the ‘Funding Information’ and ‘Financial Disclosure’ sections do not match.

When you resubmit, please ensure that you provide the correct grant numbers for the awards you received for your study in the ‘Funding Information’ section.

3. Thank you for stating the following financial disclosure:

“The research is funded by the Deanship of Research and Graduate Studies at King Khalid University under the Large Research Project under grant number RGP2/121/46”

Please state what role the funders took in the study.  If the funders had no role, please state: "The funders had no role in study design, data collection and analysis, decision to publish, or preparation of the manuscript."

If this statement is not correct you must amend it as needed.

Please include this amended Role of Funder statement in your cover letter; we will change the online submission form on your behalf.

4. Thank you for stating the following in the Acknowledgments Section of your manuscript:

“The authors extend their appreciation to the Deanship of Research and Graduate Studies at King Khalid University for funding this work through the Large Research Project under grant number RGP2/121/46.”

We note that you have provided funding information that is not currently declared in your Funding Statement. However, funding information should not appear in the Acknowledgments section or other areas of your manuscript. We will only publish funding information present in the Funding Statement section of the online submission form.

Please remove any funding-related text from the manuscript and let us know how you would like to update your Funding Statement. Currently, your Funding Statement reads as follows:

“The research is funded by the Deanship of Research and Graduate Studies at King Khalid University under the Large Research Project under grant number RGP2/121/46”

Please include your amended statements within your cover letter; we will change the online submission form on your behalf.

5. Please note that funding information should not appear in any section or other areas of your manuscript. We will only publish funding information present in the Funding Statement section of the online submission form. Please remove any funding-related text from the manuscript.

6. In the online submission form you indicate that your data is not available for proprietary reasons and have provided a contact point for accessing this data. Please note that your current contact point is a co-author on this manuscript. According to our Data Policy, the contact point must not be an author on the manuscript and must be an institutional contact, ideally not an individual. Please revise your data statement to a non-author institutional point of contact, such as a data access or ethics committee, and send this to us via return email. Please also include contact information for the third party organization, and please include the full citation of where the data can be found.

7. We note that Figure 1, 3, 4, 5, 6, 7, 8, and 9 in your submission contain map images which may be copyrighted. All PLOS content is published under the Creative Commons Attribution License (CC BY 4.0), which means that the manuscript, images, and Supporting Information files will be freely available online, and any third party is permitted to access, download, copy, distribute, and use these materials in any way, even commercially, with proper attribution. For these reasons, we cannot publish previously copyrighted maps or satellite images created using proprietary data, such as Google software (Google Maps, Street View, and Earth). For more information, see our copyright guidelines: http://journals.plos.org/plosone/s/licenses-and-copyright

We require you to either (1) present written permission from the copyright holder to publish these figures specifically under the CC BY 4.0 license, or (2) remove the figures from your submission:

1. You may seek permission from the original copyright holder of Figure1, 3, 4, 5, 6, 7, 8, and 9 to publish the content specifically under the CC BY 4.0 license.

We recommend that you contact the original copyright holder with the Content Permission Form (http://journals.plos.org/plosone/s/file?id=7c09/content-permission-form.pdf) and the following text:

“I request permission for the open-access journal PLOS ONE to publish XXX under the Creative Commons Attribution License (CCAL) CC BY 4.0 (http://creativecommons.org/licenses/by/4.0/). Please be aware that this license allows unrestricted use and distribution, even commercially, by third parties. Please reply and provide explicit written permission to publish XXX under a CC BY license and complete the attached form.”

Please upload the completed Content Permission Form or other proof of granted permissions as an "Other" file with your submission.

In the figure caption of the copyrighted figure, please include the following text: “Reprinted from [ref] under a CC BY license, with permission from [name of publisher], original copyright [original copyright year].”

2. If you are unable to obtain permission from the original copyright holder to publish these figures under the CC BY 4.0 license or if the copyright holder’s requirements are incompatible with the CC BY 4.0 license, please either i) remove the figure or ii) supply a replacement figure that complies with the CC BY 4.0 license. Please check copyright information on all replacement figures and update the figure caption with source information. If applicable, please specify in the figure caption text when a figure is similar but not identical to the original image and is therefore for illustrative purposes only.

The following resources for replacing copyrighted map figures may be helpful:

USGS National Map Viewer (public domain): http://viewer.nationalmap.gov/viewer/

The Gateway to Astronaut Photography of Earth (public domain): http://eol.jsc.nasa.gov/sseop/clickmap/

Maps at the CIA (public domain): https://www.cia.gov/library/publications/the-world-factbook/index.html and https://www.cia.gov/library/publications/cia-maps-publications/index.html

NASA Earth Observatory (public domain): http://earthobservatory.nasa.gov/

Landsat: http://landsat.visibleearth.nasa.gov/

USGS EROS (Earth Resources Observatory and Science (EROS) Center) (public domain): http://eros.usgs.gov/#

Natural Earth (public domain): http://www.naturalearthdata.com/

8. If the reviewer comments include a recommendation to cite specific previously published works, please review and evaluate these publications to determine whether they are relevant and should be cited. There is no requirement to cite these works unless the editor has indicated otherwise.

Additional Editor Comments:

**Please see the reviewer's comment below.**

Reviewer's Responses to Questions

**Comments to the Author**

1. Is the manuscript technically sound, and do the data support the conclusions?

Reviewer #1: Partly

**********

2. Has the statistical analysis been performed appropriately and rigorously?

Reviewer #1: No

**********

3. Have the authors made all data underlying the findings in their manuscript fully available?

The PLOS Data policy

Reviewer #1: No

**********

4. Is the manuscript presented in an intelligible fashion and written in standard English?

Reviewer #1: Yes

**********

Reviewer #1: Abstract

i. The abstract describes the study as a “comprehensive spatiotemporal analysis”; however, most results are based on area change statistics and visual interpretation, and the use of quantitative spatial metrics is limited.

ii. Several statements in the abstract regarding environmental impacts (e.g., pollution, habitat fragmentation, and environmental stress) are not directly supported by quantitative environmental indicators and should therefore be more cautiously phrased.

iii. The abstract refers to a “robust methodological framework,” but some important methodological limitations (e.g., simplified statistical modelling and validation procedures) are not noted, which may give the impression of overly strong analytical rigor.

Section-wise Comment

2.4. Image Classification and Accuracy Assessment

i. The derivation of spectral threshold values is not sufficiently explained; the lack of a clear calibration or optimization procedure limits reproducibility and raises concerns about methodological robustness.

ii. Several thresholds appear inconsistent or physically unrealistic (e.g., NDVI < 0.2 for vegetation; Blue < 0 for urban), which directly affects the validity of the classification results.

iii. The separation between urban and bare land remains weakly justified despite being a critical problem in arid environments with high spectral overlap.

iv. The validation sample size description is unclear, as 450 points are reported for both larger cities and for Makkah and Madinah, requiring clarification of the sampling design.

2.5. Change Detection Analysis

i. Although landscape metrics are claimed, the manuscript does not explicitly report or present them in the results, thereby limiting transparency and analytical value in this section.

ii. Change detection remains focused on gross area differences, with limited demonstration of how spatial metrics contribute to interpreting urban morphology or sprawl dynamics.

2.6. Topographic Analysis and Statistical Modelling

i. The logistic regression model is restricted to elevation and slope, oversimplifying urban growth processes and omitting key explanatory variables such as proximity to roads, population density, and economic activity.

ii. Model validation is not sufficiently detailed (e.g., no reporting of goodness-of-fit measures, ROC/AUC, or predictive accuracy), making it difficult to assess model performance.

iii. The interpretation of regression coefficients is sometimes inconsistent with reported p-values (e.g., non-significant coefficients discussed as meaningful).

2.7. Quality Control and Validation

i. Quality control procedures are largely qualitative and rely heavily on visual inspection and manual correction, introducing subjectivity and limiting reproducibility.

ii. Claims of “cross-validation” and “temporal consistency checks” are not operationally defined, and no quantitative evidence is provided to demonstrate their effectiveness.

3.1. Classification Accuracy Assessment

i. The presence of multiple near-perfect or perfect accuracy values (precision/recall = 1.0) raises concerns about optimistic bias and a lack of independence between the training and validation data.

ii. The analysis remains descriptive, with no critical discussion of class-specific confusion patterns, especially between urban and barren land.

iii. Reporting both confusion-matrix metrics and machine-learning metrics (precision/recall/F1) is not justified conceptually.

3.2. Urban Expansion Patterns and Dynamics

i. The urban expansion analysis is largely descriptive and lacks quantitative spatial or statistical metrics, limiting the analytical depth of the “spatiotemporal” claims.

ii. Urban growth is not normalized by population or density, restricting meaningful inter-city comparisons of urbanization intensity.

iii. Spatial pattern interpretation relies mainly on visual inspection, without formal spatial indicators of sprawl or compactness.

3.3. Topographic Influences on Urban Development

i. The statistical analysis does not adequately reconcile non-significant p-values with strong interpretative claims.

ii. The exclusive focus on topography ignores well-established socioeconomic drivers of urban expansion, limiting explanatory power.

3.4. Environmental Implications of Urban Expansion

i. Environmental impacts (heat islands, biodiversity loss, water stress) are asserted but not quantitatively measured, making conclusions largely inferential.

ii. No environmental indicators (e.g., LST, NDVI loss, runoff proxies) are actually computed despite strong claims.

4. Discussion

i. The discussion overinterprets results relative to the analytical depth of the methods, particularly regarding policy and sustainability implications.

ii. Many causal claims (oil revenues, migration, planning policy) are not empirically tested within the study.

5. Conclusion

i. The conclusion overstates the methodological rigor and environmental inference relative to the actual analytical framework.

ii. Claims of “robust spatiotemporal analysis” and “environmental impact assessment” are not fully supported by the presented quantitative methods.

Overall Fundamental Issue

There is a systematic mismatch between the paper’s claims (advanced spatiotemporal and environmental analysis) and the actual analytical depth of the methods, which remain largely descriptive, topography-limited, and weakly validated.

The authors are encouraged to revise the manuscript in line with the above methodological and analytical concerns. The manuscript is generally well written and intelligible; however, minor grammatical and stylistic issues are present and should be carefully revised to improve clarity and consistency.

**********

what does this mean?). If published, this will include your full peer review and any attached files.

If you choose “no”, your identity will remain anonymous but your review may still be made public.

**Do you want your identity to be public for this peer review?**  For information about this choice, including consent withdrawal, please see our Privacy Policy

Reviewer #1: No

**********

[NOTE: If reviewer comments were submitted as an attachment file, they will be attached to this email and accessible via the submission site. Please log into your account, locate the manuscript record, and check for the action link "View Attachments". If this link does not appear, there are no attachment files.]

To ensure your figures meet our technical requirements, please review our figure guidelines: https://journals.plos.org/plosone/s/figures

You may also use PLOS’s free figure tool, NAAS, to help you prepare publication quality figures: https://journals.plos.org/plosone/s/figures#loc-tools-for-figure-preparation.

NAAS will assess whether your figures meet our technical requirements by comparing each figure against our figure specifications.

# Author comment: Author response to Decision Letter 1

**DOI:** 10.1371/journal.pone.0354154.r002

1 Jun 2026

Response to Reviewers

Manuscript ID: PONE-D-25-42644

Spatiotemporal Analysis of Urban Expansion and Environmental Impacts in Five Saudi Arabian Cities by Utilizing Geospatial and Remote Sensing Approach

Dear Dr. Laxmi Kant Sharma,

Thank you for the opportunity to revise our manuscript. We appreciate the constructive feedback from the Academic Editor and the Reviewer, which has helped us improve the quality and clarity of our work. Below, we provide point-by-point responses to all comments raised, along with descriptions of the corresponding revisions made in the manuscript.

Journal Requirements

Requirement 1: Please ensure that your manuscript meets PLOS ONE's style requirements, including those for file naming.

Response: The manuscript has been reformatted to comply with PLOS ONE style requirements. File naming conventions have been followed for all submitted files.

Revision: Throughout the manuscript. Citation format converted to Vancouver numbered style per PLOS ONE guidelines.

Requirement 2: We note that the grant information you provided in the 'Funding Information' and 'Financial Disclosure' sections do not match.

Response: We have ensured that the grant number RGP2/477/46 from King Khalid University is consistently reported. The corrected funding statement is included in the cover letter for updating the online submission form.

Revision: Cover letter and online submission form.

Requirement 3: Please state what role the funders took in the study.

Response: The following statement has been prepared for the cover letter: "The funders had no role in study design, data collection and analysis, decision to publish, or preparation of the manuscript."

Revision: Cover letter.

Requirements 4 & 5: Funding information should not appear in the Acknowledgments section or other areas of your manuscript. Please remove any funding-related text from the manuscript.

Response: All funding-related text has been removed from the manuscript body, including from the Acknowledgements and Funding sections. The Acknowledgments section now reads: "The authors acknowledge the technical support provided by King Khalid University." The Funding section now directs to the online submission form.

Revision: Acknowledgements section and Funding section of the manuscript.

Requirement 6: Your current contact point is a co-author on this manuscript. Please revise your data statement to a non-author institutional point of contact.

Response: The Data Availability statement has been revised. Landsat imagery and SRTM elevation data sources are now explicitly cited as publicly available. Processed datasets are available through the Deanship of Research and Graduate Studies at King Khalid University, which serves as the institutional point of contact.

Revision: Data Availability section.

Requirement 7: Figures 1, 3, 4, 5, 6, 7, 8, and 9 contain map images which may be copyrighted.

Response: All maps in this study were generated by the authors using publicly available datasets. Land cover classification maps (Figures 3 to 7) were derived from Landsat imagery obtained from USGS Earth Explorer (public domain). Topographic maps (Figures 8 and 9) were derived from the NASA SRTM digital elevation model (public domain). Figure 1 uses administrative boundaries from Natural Earth (public domain). No proprietary or copyrighted basemaps (e.g., Google Maps, Google Earth) were used. All figure captions have been updated to state the data sources explicitly.

Revision: Captions of Figures 1, 3, 4, 5, 6, 7, 8, and 9.

Requirement 8: If the reviewer comments include a recommendation to cite specific previously published works, please review and evaluate these publications.

Response: The reviewer did not recommend specific publications for citation. No action was required on this point.

Revision: No changes needed.

Reviewer #1

Abstract

Comment i: The abstract describes the study as a "comprehensive spatiotemporal analysis"; however, most results are based on area change statistics and visual interpretation, and the use of quantitative spatial metrics is limited.

Response: We agree with this observation. The term "comprehensive" has been removed from the abstract, and the study is now described as a "spatiotemporal analysis." Similarly, "advanced" has been removed from references to remote sensing methodologies throughout the manuscript to better reflect the scope of the analysis.

Revision: Abstract (lines 48 to 52) and Introduction (line 206 and 209).

Comment ii: Several statements in the abstract regarding environmental impacts (e.g., pollution, habitat fragmentation, and environmental stress) are not directly supported by quantitative environmental indicators and should therefore be more cautiously phrased.

Response: We acknowledge that environmental indicators such as land surface temperature, NDVI loss, or runoff proxies were not directly computed. All environmental claims in the abstract have been reframed as inferred consequences consistent with the literature rather than measured outcomes. The revised text now reads: "The observed urban expansion patterns are consistent with documented effects of government policies, oil-driven economic growth, and population migration on Saudi urban landscapes, with potential implications for habitat continuity, local environmental quality, and resource pressures."

Revision: Abstract (lines 76 to 81).

Comment iii: The abstract refers to a "robust methodological framework," but some important methodological limitations are not noted, which may give the impression of overly strong analytical rigor.

Response: The phrase "robust methodological framework" has been replaced with "replicable methodological approach." Model fit statistics (Nagelkerke R² and ROC AUC ranges) have been added to the abstract to provide the reader with an immediate sense of the model's explanatory power and its limitations.

Revision: Abstract (lines 72 to 84).

Section 2.4: Image Classification and Accuracy Assessment

Comment i: The derivation of spectral threshold values is not sufficiently explained; the lack of a clear calibration or optimization procedure limits reproducibility.

Response: A new paragraph has been added to Section 2.4 describing the threshold derivation procedure. Thresholds were derived through iterative visual interpretation and spectral signature sampling from known training areas, informed by published threshold ranges for arid environments. The hierarchical decision-tree classification logic is now explicitly described, including the sequential masking approach in which spectrally distinct classes were classified first.

Revision: Section 2.4, new paragraph after the description of city-specific thresholds (lines 404 to 425).

Comment ii: Several thresholds appear inconsistent or physically unrealistic (e.g., NDVI < 0.2 for vegetation; Blue < 0 for urban).

Response: Both errors have been corrected. The vegetation NDVI threshold has been corrected from "NDVI < 0.2" to "NDVI > 0.2" (a sign error in the original table). The urban Blue threshold has been corrected from "Blue < 0" (physically impossible for DN values) to "Blue > 45 and Blue < 95," which represents the moderate reflectance range characteristic of impervious surfaces in arid environments, situated between dark water/shadow and bright desert sand.

Revision: Table 3.

Comment iii: The separation between urban and bare land remains weakly justified despite being a critical problem in arid environments.

Response: The revised Section 2.4 now includes a dedicated explanation of how urban and bare land were distinguished using the combined SAVI, NDWI, and visible band thresholds in Table 3. The text explicitly describes the spectral logic (lower blue reflectance, slightly negative SAVI, characteristic NDWI range) and acknowledges that residual confusion between these classes remains a limitation, particularly in peri-urban transition zones.

Revision: Section 2.4 (lines 415 to 425).

Comment iv: The validation sample size description is unclear, as 450 points are reported for both larger cities and for Makkah and Madinah.

Response: The text has been revised to clarify that 450 validation points were generated for each city. Sample size was determined based on minimum statistical requirements for four-class accuracy assessment (at least 50 samples per class) rather than proportional to city area.

Revision: Section 2.4 (lines 429 to 433).

Section 2.5: Change Detection Analysis

Comment i: Although landscape metrics are claimed, the manuscript does not explicitly report or present them in the results.

Response: We acknowledge this inconsistency. The claim that "fragmentation indices, connectivity measures, and shape complexity metrics were calculated" has been removed entirely, as these metrics were not reported in the results. The section now accurately describes the change detection as post-classification comparison with area-based statistics and qualitative morphological characterization. The McGarigal (2014) reference has also been removed from the reference list as it is no longer cited.

Revision: Section 2.5 (lines 489 to 494). McGarigal (2014) removed from the reference list.

Comment ii: Change detection remains focused on gross area differences, with limited demonstration of how spatial metrics contribute to interpreting urban morphology.

Response: The revised Section 2.5 now honestly describes the spatial analysis as qualitative characterization of directional expansion trends, development density patterns, and urban form in relation to topographic constraints. This framing accurately represents the analytical approach without overclaiming quantitative spatial metrics.

Revision: Section 2.5 (lines 489 to 494).

Section 2.6: Topographic Analysis and Statistical Modelling

Comment i: The logistic regression model is restricted to elevation and slope, oversimplifying urban growth processes and omitting key explanatory variables.

Response: We agree that the model is intentionally limited in scope. A new paragraph has been added to Section 2.6 explicitly acknowledging that the model was restricted to topographic variables to isolate the influence of physical terrain on development patterns, and that socioeconomic factors (proximity to roads, population density, economic activity) are recognized as important but were beyond the scope of this topography-focused analysis.

Revision: Section 2.6 (lines 524 to 532).

Comment ii: Model validation is not sufficiently detailed (e.g., no reporting of goodness-of-fit measures, ROC/AUC, or predictive accuracy).

Response: Model fit statistics have been added. Nagelkerke pseudo-R² values (ranging from 0.06 for Dammam to 0.24 for Jeddah) and ROC AUC values (0.56 to 0.72) are now reported in the revised Table 7 and discussed in Sections 3.3.2, 4.2, and the Conclusion. These values confirm modest but meaningful explanatory power for a topography-only model.

Revision: Table 7 (new columns added), Section 2.6 (lines 521 to 523), Section 3.3.2, Section 4.2, and Conclusion.

Comment iii: The interpretation of regression coefficients is sometimes inconsistent with reported p-values (e.g., non-significant coefficients discussed as meaningful).

Response: This was a critical error in the original manuscript. The entire Section 3.3.2 narrative and Section 4.2 discussion have been rewritten to accurately reflect the corrected Table 7 values. Slope is now correctly reported as significant in Jeddah (p = 0.003) and Makkah (p = 0.030), marginal in Madinah (p = 0.060), and non-significant in Riyadh (p = 0.825) and Dammam (p = 0.434). No non-significant coefficients are discussed as meaningful in the revised text.

Revision: Table 7 (corrected values), Section 3.3.2 (fully rewritten), and Section 4.2 (fully rewritten).

Section 2.7: Quality Control and Validation

Comment i: Quality control procedures are largely qualitative and rely heavily on visual inspection and manual correction.

Response: The revised Section 2.7 now describes the three quality control procedures in operational terms: visual comparison against high-resolution imagery, independent verification of uncertain classifications, and temporal plausibility screening for physically impossible transitions. The text explicitly acknowledges that these procedures are predominantly qualitative and that their subjectivity is a limitation.

Revision: Section 2.7 (lines 536 to 549).

Comment ii: Claims of "cross-validation" and "temporal consistency checks" are not operationally defined.

Response: The terms "cross-validation" and "temporal consistency checks" have been replaced with specific operational descriptions of the procedures actually performed, as described above.

Revision: Section 2.7 (lines 536 to 549).

Section 3.1: Classification Accuracy Assessment

Comment i: The presence of multiple near-perfect or perfect accuracy values (precision/recall = 1.0) raises concerns about optimistic bias.

Response: A new paragraph has been added explaining that perfect values occur primarily for water body classification due to strong spectral separability in SWIR bands. The text acknowledges that spatial proximity of training and validation samples within homogeneous spectral environments may contribute to optimistic accuracy estimates, a recognized challenge in remote sensing validation.

Revision: Section 3.1 (lines 592 to 608).

Comment ii: The analysis remains descriptive, with no critical discussion of class-specific confusion patterns, especially between urban and barren land.

Response: The new paragraph in Section 3.1 now explicitly discusses urban-barren confusion patterns, noting that this confusion is most prevalent in peri-urban transition zones where bright urban materials exhibit spectral similarity to surrounding sandy surfaces.

Revision: Section 3.1 (lines 597 to 604).

Comment iii: Reporting both confusion-matrix metrics and machine-learning metrics (precision/recall/F1) is not justified conceptually.

Response: The text accompanying Table 5 now provides justification: precision corresponds to user's accuracy and recall to producer's accuracy in the remote sensing context, and these metrics are presented in machine-learning notation to facilitate comparison with studies using automated classification approaches. Alternatively, Table 5 can be moved to Supplementary Material if the editor prefers.

Revision: Section 3.1, text preceding Table 5 (lines 792 to 802).

Section 3.2: Urban Expansion Patterns and Dynamics

Comment i: The urban expansion analysis is largely descriptive and lacks quantitative spatial or statistical metrics.

Response: We acknowledge the descriptive nature of the spatial pattern analysis. References to "spatiotemporal" analysis have been moderated throughout the manuscript. Annual growth rates and growth percentages have been added to Table 6. Qualitative morphological descriptions are now explicitly framed as such.

Revision: Table 6 (new columns), Section 3.2 text.

Comment ii: Urban growth is not normalized by population or density.

Response: Population-normalized analysis has been added using census data from 1992 and 2022 (General Authority for Statistics, Kingdom of Saudi Arabia). Table 6 now includes population figures, per-capita urban area for both time periods, annual growth rates, and population growth percentages. A new paragraph in Section 3.2.1 discusses the sprawl vs. densification patterns revealed by this comparison: Riyadh and Jeddah show sprawl (area growth exceeding population growth), while Makkah and Dammam show densification (population growth exceeding area growth).

Revision: Table 6 (enhanced with population columns), Section 3.2.1 (new paragraph, lines 1218 to 1233). New reference added: General Authority for Statistics, Kingdom of Saudi Arabia.

Comment iii: Spatial pattern interpretation relies mainly on visual inspection, without formal spatial indicators of sprawl or compactness.

Response: The revised text acknowledges that spatial pattern characterization is qualitative and based on visual interpretation of classified maps. This is now framed appropriately as qualitative assessment rather than quantitative spatial analysis. Future research incorporating formal compactness or sprawl indices is recommended in the limitations section.

# Editor report: Decision Letter 1

**DOI:** 10.1371/journal.pone.0354154.r003

**Authors:** Laxmi Kant Sharma

6 Jul 2026

<p>Spatiotemporal Analysis of Urban Expansion and Environmental Impacts in Five Saudi Arabian Cities by Utilizing Geospatial and Remote Sensing Approach

PONE-D-25-42644R1

Dear Dr. Abdulla ,

We’re pleased to inform you that your manuscript has been judged scientifically suitable for publication and will be formally accepted for publication once it meets all outstanding technical requirements.

Within one week, you’ll receive an e-mail detailing the required amendments. When these have been addressed, you’ll receive a formal acceptance letter and your manuscript will be scheduled for publication.

An invoice will be generated when your article is formally accepted. Please note, if your institution has a publishing partnership with PLOS and your article meets the relevant criteria, all or part of your publication costs will be covered. Please make sure your user information is up-to-date by logging into Editorial Manager at Editorial Manager® and clicking the ‘Update My Information' link at the top of the page. For questions related to billing, please contact billing support.

If your institution or institutions have a press office, please notify them about your upcoming paper to help maximize its impact. If they’ll be preparing press materials, please inform our press team as soon as possible -- no later than 48 hours after receiving the formal acceptance. Your manuscript will remain under strict press embargo until 2 pm Eastern Time on the date of publication. For more information, please contact onepress@plos.org.

Kind regards,

Laxmi Kant Sharma, PhD

Academic Editor

PLOS One

Additional Editor Comments (optional):

The revised manuscript may now be accepted for publication.

Reviewers' comments:

# Editor report: Acceptance letter

**DOI:** 10.1371/journal.pone.0354154.r004

**Authors:** Laxmi Kant Sharma

PONE-D-25-42644R1

PLOS One

Dear Dr. Kafy,

I'm pleased to inform you that your manuscript has been deemed suitable for publication in PLOS One. Congratulations! Your manuscript is now being handed over to our production team.

At this stage, our production department will prepare your paper for publication. This includes ensuring the following:

* All references, tables, and figures are properly cited

* All relevant supporting information is included in the manuscript submission,

* There are no issues that prevent the paper from being properly typeset

You will receive further instructions from the production team, including instructions on how to review your proof when it is ready. Please keep in mind that we are working through a large volume of accepted articles, so please give us a few days to review your paper and let you know the next and final steps.

Lastly, if your institution or institutions have a press office, please let them know about your upcoming paper now to help maximize its impact. If they'll be preparing press materials, please inform our press team within the next 48 hours. Your manuscript will remain under strict press embargo until 2 pm Eastern Time on the date of publication. For more information, please contact onepress@plos.org.

You will receive an invoice from PLOS for your publication fee after your manuscript has reached the completed accept phase. If you receive an email requesting payment before acceptance or for any other service, this may be a phishing scheme. Learn how to identify phishing emails and protect your accounts at https://explore.plos.org/phishing.

If we can help with anything else, please email us at customercare@plos.org.

Thank you for submitting your work to PLOS One and supporting open access.

Kind regards,

PLOS ONE Editorial Office Staff

on behalf of

Dr. Laxmi Kant Sharma

Academic Editor

PLOS One
